# Supervised maximum-likelihood weighting of composite protein networks for complex prediction

**DOI:** 10.1186/1752-0509-6-S2-S13

**Published:** 2012-12-12

**Authors:** Chern Han Yong, Guimei Liu, Hon Nian Chua, Limsoon Wong

**Affiliations:** 1 Graduate School for Integrative Sciences and Engineering, National University of Singapore, Singapore; 2 School of Computing, National University of Singapore, Singapore; 3 Institute for Infocomm Research, Singapore

## Abstract

**Background:**

Protein complexes participate in many important cellular functions, so finding the set of existent complexes is essential for understanding the organization and regulation of processes in the cell. With the availability of large amounts of high-throughput protein-protein interaction (PPI) data, many algorithms have been proposed to discover protein complexes from PPI networks. However, such approaches are hindered by the high rate of noise in high-throughput PPI data, including spurious and missing interactions. Furthermore, many transient interactions are detected between proteins that are not from the same complex, while not all proteins from the same complex may actually interact. As a result, predicted complexes often do not match true complexes well, and many true complexes go undetected.

**Results:**

We address these challenges by integrating PPI data with other heterogeneous data sources to construct a composite protein network, and using a supervised maximum-likelihood approach to weight each edge based on its posterior probability of belonging to a complex. We then use six different clustering algorithms, and an aggregative clustering strategy, to discover complexes in the weighted network. We test our method on *Saccharomyces cerevisiae *and *Homo sapiens*, and show that complex discovery is improved: compared to previously proposed supervised and unsupervised weighting approaches, our method recalls more known complexes, achieves higher precision at all recall levels, and generates novel complexes of greater functional similarity. Furthermore, our maximum-likelihood approach allows learned parameters to be used to visualize and evaluate the evidence of novel predictions, aiding human judgment of their credibility.

**Conclusions:**

Our approach integrates multiple data sources with supervised learning to create a weighted composite protein network, and uses six clustering algorithms with an aggregative clustering strategy to discover novel complexes. We show improved performance over previous approaches in terms of precision, recall, and number and quality of novel predictions. We present and visualize two novel predicted complexes in yeast and human, and find external evidence supporting these predictions.

## Background

Protein complexes participate in many important cellular functions, so finding the set of existent complexes is essential for understanding the mechanism, organization, and regulation of processes in the cell. Since protein complexes are groups of interacting proteins, many methods have been proposed to discover complexes from protein-protein interaction (PPI) data, which has been made available in large amounts by high-throughput experimental techniques. Typically, complexes are predicted based on topological characteristics in the PPI network. For example, many approaches search for regions of high density or connectivity [1-5]. Other approaches further incorporate subgraph diameters of known complexes [6], and core-attachment models of connected clusters [7,8]. Qi *et al. *used a set of topological features including density, degree, edge weight, and graph eigenvalues, with a supervised naive-Bayes approach to learn these feature parameters from training complexes [9].

**Figure 1:**
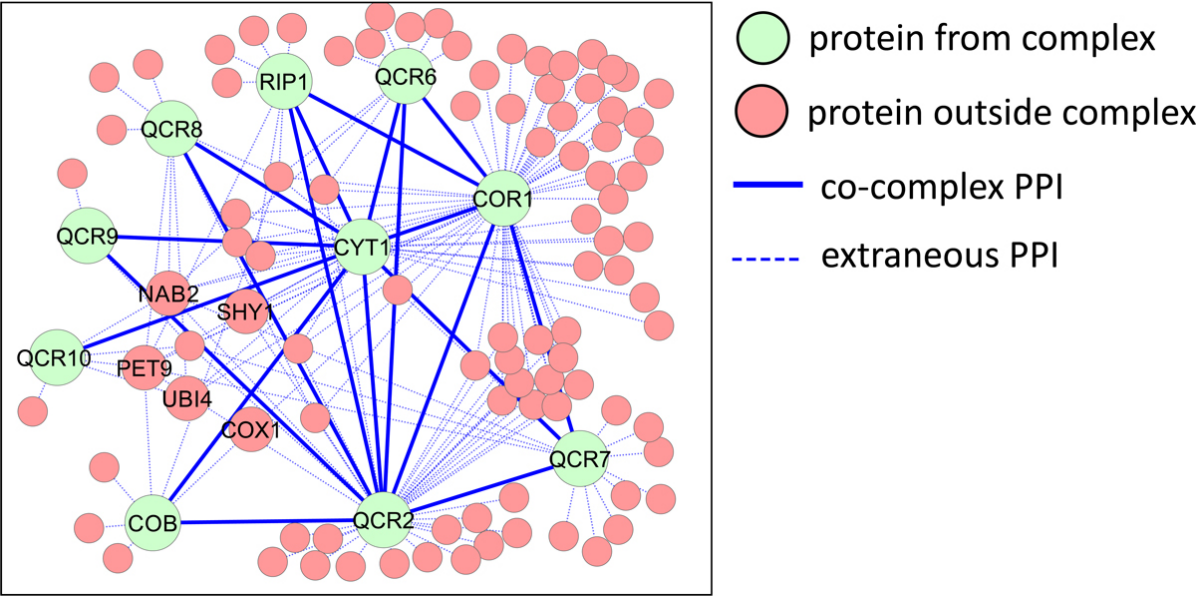
**PPI subgraph of the mitochondrial cytochrome bc1 complex**. Nineteen interactions were detected between the ten proteins from the complex, while many extraneous interactions were detected. Five example proteins from transient interactions are shown: NAB2 and UBI4 are involved in mRNA polyadenylation and protein ubiquitination, while PET9, SHY1, and COX1 are mitochondrial membrane proteins that are also involved in the electron-transport chain. The extraneous interactions around the complex makes its discovery difficult. All such network figures were generated by Cytoscape [30].

The performance of these complex discovery algorithms is reliant on the quality of the protein interaction data, which is often associated with substantial numbers of spuriously-detected interactions (false positives) and missing interactions (false negatives). Furthermore, many protein pairs that actually do interact with each other are not located in the same complex, for example, protein pairs that bind temporarily to perform a function. We refer to such interactions as transient interactions. Finally, not all proteins in the same complex may interact with each other, making its PPI subgraph far from complete. Figure 1 provides an illustrative example of these challenges. The mitochondrial cytochrome bc1 complex is a well-known complex involved in the electron-transport chain in the mitochondrial inner membrane. In *Saccharomyces cerevisiae *(yeast), this complex is composed of ten proteins. Figure 1 shows the PPI subgraph around these ten proteins, using PPI data obtained from BioGRID [10], IntAct [11] and MINT [12]. Nineteen PPIs (out of a possible 45) were detected between these ten proteins; the rest remain undetected, likely due to the difficulty of detecting interactions between membrane proteins, or because not all proteins in this complex interact with each other. 145 extraneous interactions were detected between the proteins from this complex and 94 proteins outside the complex. While some of these extraneous interactions might be spuriously detected, others constitute transient interactions. Five proteins likely involved in such transient interactions are shown: NAB2 and UBI4 are involved in mRNA polyadenylation and protein ubiquitination respectively, and bind to many proteins to perform their functions; PET9, SHY1, and COX1 are mitochondrial membrane proteins that are also involved in the electron-transport chain, and interact with proteins of the complex, although they are not part of it. The density of the complex is lost amidst the noise of the extraneous interactions, making the discovery of this complex from PPI data extremely difficult: none of the six complex discovery algorithms we use in this paper successfully detected it.

Many algorithms have been developed to assess the reliability of high-throughput protein interactions [13-15] or predict new protein interactions [16-19], using various information such as gene sequences, annotations, interacting domains, 3D structures, experimental repeatability, or topological characteristics of PPI networks. These approaches have been shown to be effective in reducing false positives or false negatives. In our previous work [3], we have shown that using the topology of the PPI network to weight interactions, remove unreliable interactions, and posit new interactions improves the performance of several complex discovery algorithms. While such approaches are effective in reducing the impact of spuriously-detected and missing interactions, they do not directly address transient interactions and non-interacting complex proteins.

Researchers have also proposed integrating heterogeneous data sources with supervised approaches to predict co-complex protein pairs (protein pairs that belong to the same complex), using a reference set of training complexes. Data integration leverages on the fact that diverse data sources other than PPI can also reveal co-complex relationships, while a supervised approach targeted at predicting co-complex protein pairs can be trained to discriminate between actual co-complex interactions and spuriously-detected or transient interactions. Qiu and Noble [20] integrated PPI, protein sequences, gene expression, interologs, and functional information, to train kernel-based models, and achieved high classification accuracy in predicting co-complex protein pairs. However, they did not apply or test their method on reconstructing and predicting complexes. Wang *et al. *[4] integrated PPI, gene expression, localization annotations, and transmembrane features, and applied a boosting method to predict co-complex protein pairs. They showed that this approach, combined with their proposed clustering method HACO, achieved higher sensitivity in recovering reference complexes compared to unsupervised approaches. However, they did not explore how well their classification approach works when used in conjunction with other clustering methods: while sensitivity was improved, many reference complexes were still unable to be predicted in part due to limitations of HACO, thus raising the question of whether other clustering methods may also see an improvement when used with their co-complex predictions. Furthermore, these approaches directly produce co-complex affinity scores between protein pairs, without providing measurements of the predictive strengths of the different data sources, nor how the different score values of each data source indicate co-complex relationships. In our view, this is important when integrating different data sources: while using PPI for complex prediction is biologically reasonable because proteins in a complex interact and bind with each other, using other data sources such as sequences, expression, or literature co-occurrence is not as biologically intuitive, even if they do reveal co-complex relationships. Providing a measurement of how these data sources contribute to co-complex predictions allows human judgment of the validity and credibility of predicted novel complexes.

We propose a method to address these challenges of complex discovery: first, the PPI network is integrated with other heterogeneous data sources that specify relationships between proteins, such as functional association and co-occurrence in literature, to form an expanded, composite network. Next, each edge is weighted based on its posterior probability of belonging to a protein complex, using a naive-Bayes maximum-likelihood model learned from a set of training complexes. A complex discovery algorithm can then be used on this weighted composite network to predict protein complexes. Our method offers several advantages over current unsupervised or non-integrative weighting approaches. First, a composite protein network constructed from multiple data sources is more likely to have denser subgraphs for protein complexes, as it not only reduces the number of missing interactions, but also adds edges between non-interacting proteins from the same complex, because such proteins are likely to be related in ways other than by physical interactions. Second, learning a model from training complexes not only provides a powerful method to assess the reliability of interactions, but also allows the discrimination between transient and co-complex interactions. Third, utilizing multiple data sources to assess the reliability of interactions is likely to be more accurate than using just PPI data.

Our choice of a naive-Bayes maximum-likelihood model also offers several advantages over other supervised data-integration approaches. Firstly our model is transparent, in that learned parameters can be validated and analyzed, for example to reveal the predictive strengths of the different data sources. Furthermore, for a predicted complex, the learned parameters can then be used to visualize the component evidences from the different data sources, allowing human judgment of the credibility of the prediction. Second, maximum-likelihood models are known to be robust and have low variance, even when few training samples are available. Although we describe our experiments using yeast and human, this is important when we apply our approach to less-studied organisms with fewer known complexes available for training. Finally, we utilize different clustering algorithms as well as a simple aggregative clustering strategy to evaluate the performance of our method, and show that we improve the performance of complex prediction compared to other weighting methods.

## Methods

### Building the composite network

Heterogeneous data sources are combined to build the composite network. Each data source provides a list of scored protein pairs: for each pair of proteins (*u, v*) with score *s, u *is related to *v *with score *s*, according to that data source. For both yeast and human, the following data sources are used:

• PPI data is obtained by taking the union of physical interactions from BioGRID [10], IntAct [11] and MINT [12] (data from all three repositories downloaded in November 2011). Interactions are scored using a topological function, Iterative AdjustCD (with two iterations), which has been shown to improve the performance of complex discovery [3]. Iterative AdjustCD uses expectation maximization to score each interaction (*u, v*) based on the number of shared neighbors of *u *and *v*. Interactions between proteins that have no shared neighbors are regarded as unreliable and are discarded. Protein pairs that do not directly interact but have shared neighbors are also scored; such pairs with scores above 0.1 are added as new interactions, and are called Level 2 or L2-PPIs. We consider PPIs and L2-PPIs as two separate data sources.

• Predicted functional association data is obtained from the STRING database [21] (data downloaded in January 2012). STRING predicts each association between two proteins *u *and *υ *(or their respective genes) using the following evidence types: gene co-occurrence across genomes; gene fusion events; gene proximity in the genome; homology; coexpression; physical interactions; co-occurrence in literature; and orthologs of the latter five evidence types transferred from other organisms (STRING also includes evidence obtained from databases, which we discard as this may include co-complex relationships which we are trying to predict). Each evidence type is associated with quantitative information (e.g. the number of gene fusion events), which STRING maps to a confidence score of functional association based on co-occurrence in KEGG pathways. The confidence scores of the different evidence types are then combined probabilistically to give a final functional association score for (*u, v*). Only pairs with score greater than 0.5 are kept.

• Co-occurrence of proteins or genes in PubMed literature (data downloaded in January 2012). Each pair (*u, υ*) is scored by the Jaccard similarity of the sets of papers that *u *and *υ *appear in:

$$s=\frac{\left|A_{u}\cap A_{v}\right|}{\left|A_{u}\cup A_{v}\right|}$$

where *A_x _*is the set of PubMed papers that contain protein *x*. For yeast, that would be the papers that contain the gene name or open reading frame (ORF) ID of *x *as well as the word "cerevisiae"; for human that would be the papers that contain the gene name or Uniprot ID of *x *as well as the words "human" or "sapiens".

While there seems to be overlap between STRING's use of PPI and literature co-occurrence data with our use of them as separate data sources, note that STRING uses these data as only as component evidences for functional association and scores them accordingly. Thus we treat the STRING data as a representation of functional association between proteins, regardless of how this association was derived. Table 1 gives some summarizing statistics for these data sources.

**Table 1:** Statistics of data sources

		YEAST			HUMAN		
**Data source**	**Description**	**# pairs**	**# distinct proteins**	**% complex edges**	**# pairs**	**# distinct proteins**	**% complex edges**

PPI	Physical protein-protein interactions	106328	5429	5.8%	48098	6285	13.9%
L2-PPI	Level 2 PPI	181175	3987	1.1%	131705	7913	5.5%
STRING	Predicted functional association	175712	5964	5.7%	311435	14784	3.1%
PubMed	Literature co-occurrence	161213	5109	4.9%	91751	10659	4.3%

All		531800	6084	2.1%	522668	17264	3.4%

In the composite network, vertices represent proteins and edges represent relationships between proteins. The composite network has an edge between proteins *u *and *v *if and only if there is a relationship between *u *and *v *according to any of the data sources.

### Edge-weighting by posterior probability

Next, each edge (*u, v*) is weighted based on its posterior probability of being a co-complex edge (i.e. both *u *and *v *are in the same complex), given the scores of the data source relationships between *u *and *v*.

We use a naive-Bayes maximum-likelihood model to derive the posterior probability. Each edge (*u, v*) between proteins *u *and *v *of the composite network is cast as a data instance. The set of features is the set of data sources, and for each instance (*u, v*), feature *F *has value *f *if proteins *u *and *υ *are related by data source *F *with score *f*. If *u *and *v *are not related by data source *F*, then feature *F *is given a score of 0. Using a reference set of protein complexes, each instance (*u, v*) in the training set is given a class label *co-complex *if both *u *and *υ *are in the same complex; otherwise its class label is *non-co-complex *Learning proceeds by two steps:

1. Minimum description length (MDL) supervised discretization [22] is performed to discretize the features. MDL discretization recursively partitions the range of each feature to minimize the information entropy of the classes. If a feature cannot be discretized, that means it is not possible to find a partition that reduces the information entropy, so the feature is removed. Thus this step also serves as simple feature selection.

2. The maximum-likelihood parameters are learned for the two classes *co-complex *and *non-co-complex*:

$$\begin{array}{c} P\left(F=f|co-comp\right)=\frac{n_{c,F=f}}{n_{c}}\\ P\left(F=f|non-co-comp\right)=\frac{n_{\neg c,F=f}}{n_{\neg c}} \end{array}$$

for each discretized value *f *of each feature *F. n_c _*is the number of edges with class label *co-complex*, $n_{c,F=f}$ is the number of edges with class label *co-complex *and whose feature *F *has value *f*, $n_{\neg c}$ is the number of edges with class label *non-co-complex*, and $n_{\neg c,F=f}$ is the number of edges with class label *non-co-complex *and whose feature *F *has value *f*

After learning the maximum-likelihood model, the weight for each edge *e *with feature values *F*_1 _= *f*_1_, *F*_2 _= *f*_2_, . . . is calculated as its posterior probability of being a co-complex edge:

$$\begin{array}{llll} & \;weight\left(e\right)\; & & \;\\ = & \;P\left(co-comp|F_{1}=f_{1},F_{2}=f_{2},\ldots \right)\; & & \;\\ = & \;\frac{P\left(F_{1}=f_{1},F_{2}=f_{2},\ldots |co-comp\right)P\left(co-comp\right)}{Z}\; & & \;\\ = & \;\frac{\prod_{i}P\left(F_{i}=f_{i}|co-comp\right)P\left(co-comp\right)}{Z}\; & & \;\\ = & \;\frac{\prod_{i}P\left(F_{i}=f_{i}|co-comp\right)P\left(co-comp\right)}{\prod_{i}P\left(F_{i}=f_{i}|co-comp\right)P\left(co-comp\right)+\prod_{i}P\left(F_{i}=f_{i}|non-co-comp\right)P\left(non-co-comp\right)}\; & & \;\\ & \; & \end{array}$$

where *Z *is a normalizing factor to ensure the probabilities sum to 1. Although the second last equality makes the assumption that the features are independent, naive-Bayes classifiers have been found to perform well even when this assumption is false [23]. Specifically, while the probability estimates are frequently inaccurate, their rank orders usually remain correct, so that edges with likelier co-complex feature values are assigned higher scores than edges with likelier non-co-complex feature values.

### Complex discovery

After the composite network is weighted, the top *k *edges are used by a clustering algorithm to predict protein complexes. We use the following clustering algorithms in our study:

**Markov Cluster Algorithm (MCL) **[1] simulates stochastic flow to enhance the contrast between regions of strong and weak flow in the graph. The process converges to a partition with a set of high-flow regions (the clusters) separated by boundaries with no flow.

**Restricted Neighborhood Search Clustering (RNSC) **[2] is a local search algorithm that explores the solution space to minimize a cost function, calculated according to the number of intra-cluster and inter-cluster edges. RNSC first composes an initial random clustering, and then iteratively moves nodes between clusters to reduce the clustering's cost. It also makes diversification moves to avoid local minima. RNSC performs several runs, and reports the clustering from the best run.

**IPCA **[6] expands clusters from seeded vertices, based on rules that encode prior knowledge of the topological structure of protein complexes' PPI subgraphs. Whether a cluster is expanded to include a vertex is determined by the diameter of the resultant cluster and the connectivity between the vertex and the cluster.

**Clustering by Maximal Cliques (CMC) **[3] first generates all the maximal cliques from a given network, and then removes or merges highly overlapping clusters based on their inter-connectivity as follows. If the overlap between two maximal cliques exceeds a threshold *overlap_thres*, then CMC checks whether the inter-connectivity between the two cliques exceeds a second threshold *merge_thres*. If it does, then the two cliques are merged; otherwise, the clique with lower density is removed.

**Hierarchical Agglomerative Clustering with Overlap (HACO) **[4] first considers all vertices as individual clusters, then iteratively merges pairs of clusters with high connectivity between them. At each merge, the two constituting clusters are remembered; when the merged cluster *A *is later merged with another cluster *B*, it also tries to merge the remembered constituting clusters of *A *with the cluster *B*, and keeps the (possibly overlapping) resultant clusters if they are highly connected.

**Clustering with Overlapping Neighborhood Expansion (ClusterONE) **[5] greedily expands clusters from seeded vertices to maximize a cohesiveness function, which is based on the edge weights within a cluster and the edge weights connecting the cluster to the rest of the network. It then merges highly-overlapping clusters.

CMC, MCL, HACO, and ClusterONE are able to utilize edge weights in their input networks, whereas RNSC and IPCA do not; in this case, the selection of the top k edges provides less noisy networks as inputs to the algorithms.

CMC, MCL, and HACO utilize parameters whose optimal values are at least partly dependent on the input networks' distribution of edge weights. For example, given an input network with high edge weights, using CMC with too low a *merge_thres *produces too many clusters consisting of merged cliques. Thus, we run these algorithms with a range of values for their respective parameters, so as to obtain a more comprehensive picture of their performances across different weighting approaches. We run ClusterONE, RNSC, and IPCA with mostly default or recommended parameters. The parameter settings used in our experiments for the six clustering algorithms are given in Table 2.

**Table 2:** Parameters for clustering algorithms

Clustering algorithm	Parameter settings
CMC	min deg ratio = 1, min size = 4, overlap thres = 0.5, merge thres = 0.25
	min deg ratio = 1, min size = 4, overlap thres = 0.5, merge thres = 0.5
	min deg ratio = 1, min size = 4, overlap thres = 0.5, merge thres = 0.75
HACO	-c c 1 -g 0.1
	-c c 1 -g 0.3
	c c 0.75 -g 0.1
MCL	-I 2
	-I 3
	-I 4
ClusterONE	-s 4 -d 0
IPCA	-S4 -P2 -T0.4
RNSC	-e10 -D50 -d10 -t20 -T3

For any cluster *C *produced by any of these clustering algorithms, we define its score as its weighted density:

$$score\left(C\right)=dens\left(C\right)=\frac{\sum_{u\in C,v\in C}w\left(u,v\right)}{\left|C\right|\;\left(\left|C\right|-1\right)}$$

We also use a simple voting-based aggregative strategy **COMBINED**, which takes the union of the clusters produced by the six algorithms above. If two or more clusters are found to be similar to each other, then only the cluster with the highest weighted density is kept, and its score is defined as its weighted density multiplied by the number of algorithms that produced the group of similar clusters; otherwise its score is its weighted density as usual. We define two clusters *C *and *D *to be similar if *Jaccard*(*C, D*) >= 0.75, where *Jaccard*(*C, D*) is the Jaccard similarity between the proteins contained in C and D:

$$Jaccard\left(C,D\right)=\frac{\left|V_{C}\cap V_{D}\right|}{\left|V_{C}\cup V_{D}\right|}$$

where *V_X _*is the set of proteins contained in *X*.

## Results

### Experimental setup

In our main experiment, we compare the performance of five weighting approaches:

1. SWC: supervised weighting of composite network (our proposed method)

2. BOOST: supervised weighting of composite network using LogitBoost [4]

3. TOPO: unsupervised topological weighting of PPI network with Iterative AdjustCD [3], including level-2 PPIs (these weights are equivalent to the PPI and L2-PPI features in our composite network)

4. STR: network of predicted and scored functional associations from STRING [21] (these weights are equivalent to the STRING feature in our composite network)

5. NOWEI: unweighted PPI network

We perform random sub-sampling cross-validation, repeated over ten rounds, using manually curated complexes as reference complexes for training and testing. For yeast, we use the CYC2008 [24] set which consists of 408 complexes. Only complexes of size greater than three proteins are used for testing; there are 149 such complexes in CYC2008. For human, we use the CORUM [25] set which consists of 1829 complexes, of which 714 are of size greater than three. In each cross-validation round, *t% *of the complexes of size greater than three are selected for testing, while all the remaining complexes are used for training. Each edge (*u, v*) in the network is given a class label *co-complex *if *u *and *v *are in the same training complex, otherwise its class label is *non-co-complex*. For SWC and BOOST, learning is performed using these labels, and the edges of the entire network are then weighted using the learned models. TOPO, STRING, and NOWEI require no learning, so the labels are not used; instead, for TOPO the edges of the network are weighted with topological scores, for STRING the edges are weighted with functional association scores, and for NOWEI all edges are given weight 1. The top-weighted *k *edges from the network are then used by the clustering algorithms to predict complexes. For NOWEI we use k=all edges, while for SWC, BOOST, TOPO, and STRING, we use k=10000,20000. We do not use all edges for these four weighting methods, because weighting enriches the network in dense clusters, which causes some of the clustering algorithms to require too much time to run when all edges are used; moreover, our experiments indicate that the performance of these methods drop when more than 20000 edges are used. The predicted clusters are evaluated on how well they match the test complexes.

We designed our experiment to simulate a real-use scenario of complex prediction in an organism where a few complexes might already be known, and novel complexes are to be predicted: in each round of cross-validation, the training complexes are those that are known and leveraged for learning to discover new complexes, while the test complexes are used to evaluate the performance of each approach at this task. Thus we use a large percentage of test complexes t=90%. In yeast, this gives 134 test complexes (among the 149 complexes of size greater than three), and 274 training complexes (only 15 of size greater than three); in human, this gives 643 test complexes (among the 714 of size greater than three), and 1186 training complexes (71 of size greater than three).

### Evaluation methods

to evaluate the predicted clusters. First, a cluster *P *is said to match a complex *C *at a given match threshold *match_thres *if *Jaccard*(*P, C*) >= *match_thres*. Each cluster *C *is ranked by its score. To obtain a precision-recall graph, we calculate and plot the precision and recall of the predicted clusters at various cluster-score thresholds. Given a set of predicted clusters $P=\left\{P_{1},\;P_{2},\;\ldots \right\}$, a set of test reference complexes $C=\left\{C_{1},\;C_{2},\;\ldots \right\}$, and a set of training reference complexes $T=\left\{T_{1},\;T_{2},\;\ldots \right\}$, the recall and precision at score threshold *d *are defined as follows:

$$\begin{array}{c} Recall_{d}=\\ \frac{\left|\left\{C_{i}|C_{i}\in C\wedge \exists P_{j}\in P,dens\left(P_{j}\right)\geq d,P_{j}matchesC_{i}\right\}\right|}{\left|C\right|}\\ Precision_{d}=\\ \frac{\left|\left\{P_{j}|P_{j}\in P,dens\left(P_{j}\right)\geq d\wedge \exists C_{i}\in C,C_{i}matchesP_{j}\right\}\right|}{\left|\left\{P_{k}|P_{k}\in P,dens\left(P_{k}\right)\geq d\wedge \left(∄T_{i}\in T,T_{i}matchesP_{k}\vee \exists C_{i}\in C,C_{i}matchesP_{k}\right)\right\}\right|} \end{array}$$

The precision of clusters is calculated only among those clusters that do not match a training complex, to eliminate the bias of the supervised approaches (SWC and BOOST) for predicting training complexes well. The precision-recall area under curve (AUC) is used as a summarizing statistic for each method's performance. Besides evaluating the performance of complex prediction, we also evaluate the performance of edge classification, in which the edge weights are used to classify edges as co-complex or non-co-complex edges.

To evaluate the quality of novel predicted complexes, we define three measures of semantic coherence for each complex: its biological process (BP), cellular compartment (CC), and molecular function (MF) semantic coherence. These are calculated from the proteins' annotations to Gene Ontology (GO) terms, which span the three classes BP, CC, and MF [26]. We use the most informative common ancestor method of calculating the semantic similarity between two proteins, as outlined in [27]. Briefly, the semantic similarity of two GO terms is first defined as the information content of their most informative common ancestor. Next, the BP semantic similarity of two proteins is defined as the highest semantic similarity between their two sets of annotated BP terms. Then, we define the BP semantic coherence of a predicted complex as the average BP semantic similarity between every pair of proteins in that complex (likewise for CC and MF).

### Classification of co-complex edges

#### Yeast

We first evaluate each approach in classification of co-complex edges. Here, each weighting approach is used to weight the network edges, and the edges are classified as co-complex by taking a threshold on their weights. We obtain precision-recall graphs (solid markers, left axis) by taking a series of decreasing thresholds; at each recall level, we also indicate the proportion of test complexes covered by at least one predicted edge (hollow markers, right axis).

**Figure 2:**
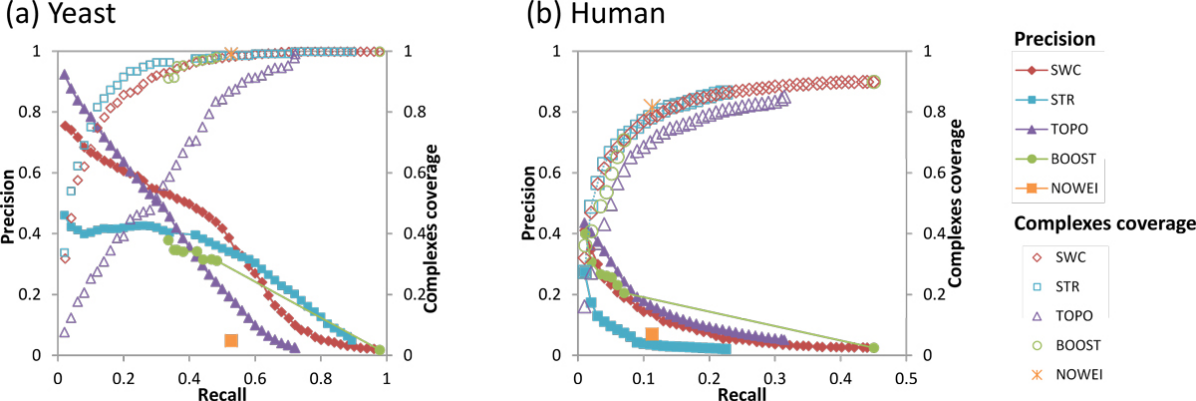
**Precision-recall graph for classification of co-complex edges using the five weighting schemes**. (a) Classification of yeast co-complex edges. SWC and BOOST achieve the highest recall through data integration. TOPO has high precision for its top-scoring edges, but these are clustered in a few complexes. SWC achieves higher precision than STR, except when too many edges are considered. BOOST classifies edges categorically, giving high scores to one set of edges with about 50% recall and 35% precision, and low scores to the remainder. (b) Classification of human co-complex edges. Recall and precision for human is much lower than for yeast. TOPO has higher precision than SWC, but its predicted edges are clustered in fewer complexes. BOOST classifies edges categorically, and its high-scoring edges achieve 7% recall, with comparable precision with SWC. NOWEI has slightly higher precision than STR, which has the lowest precision.

Figure 2a shows the performance of the five weighting approaches for classification of co-complex edges in yeast, and demonstrates that SWC achieves decent precision levels, while covering a large number of complexes. Compared to TOPO, SWC has lower precision among the highly-weighted edges, indicating that edges with high topological scores are likelier to be co-complex compared to edges with high SWC scores. However, these edges are clustered in a few test complexes, giving lower complex coverage. When more edges are included to predict co-complex edges in a wider range of complexes, TOPO's precision drops well below that of SWC. Thus, topological weighting can only accurately predict edges in a few densely-connected complexes whose edges have high topological scores; for less dense complexes, SWC performs better by using multiple data sources and supervised learning.

On the other hand, SWC is more accurate than STRING in predicting co-complex edges with high weights, because many proteins that are highly functionally associated are not co-complex, while SWC's supervised learning approach produces weights that are targeted at predicting co-complex edges, so highly-weighted edges are likelier to be co-complex. However, to retrieve even more co-complex edges by lowering the weight threshold, STRING's precision rises above SWC's, indicating that finding co-complex edges in this region might be better served simply by functional association.

BOOST integrates the same data sources as SWC, but uses LogitBoost instead to learn to classify co-complex edges. Its points in the graph are clustered in two regions: one set of edges are given high scores, achieving about 40% recall and 35% precision (lower than SWC's precision of 50% at this recall level), while the remaining edges are given low scores. Thus BOOST performs classification in a categorical manner, whereas SWC produces co-complex scores that reflect a wide range of confidence.

Finally, the performance of NOWEI, which uses unweighted PPI edges, appears as a single point on the graph, and shows that the PPI edges cover only 53% of co-complex edges, with a precision of 5%.

#### Human

Figure 2b shows the corresponding precision-recall graphs for classification of co-complex edges in human. Compared to yeast, the coverage of co-complex edges is much lower in human.

Compared to TOPO, SWC has lower precision along TOPO's entire recall range. However, once again TOPO's predicted edges are clustered in fewer complexes, giving lower complex coverage: for example, to cover 80% of complexes requires TOPO to recall 22% of edges at a precision of 8%; SWC has to recall only 13% of edges at a higher precision of 11% to cover the same amount of complexes. Thus, for human as well as yeast, SWC is able to predict co-complex edges for a wider range of complexes compared to TOPO, whose range is limited to fewer complexes that are densely connected.

For human, STRING's functional association scores are the least accurate for predicting co-complex edges, giving the lowest precision among all the weighting approaches.

Just like in yeast, BOOST performs classification in a categorical manner: a set of edges are predicted as co-complex with high scores, achieving 7% recall and similar precision levels as SWC, while the remaining edges are predicted as non-co-complex with low scores.

### Prediction of complexes

#### Yeast

**Figure 3:**
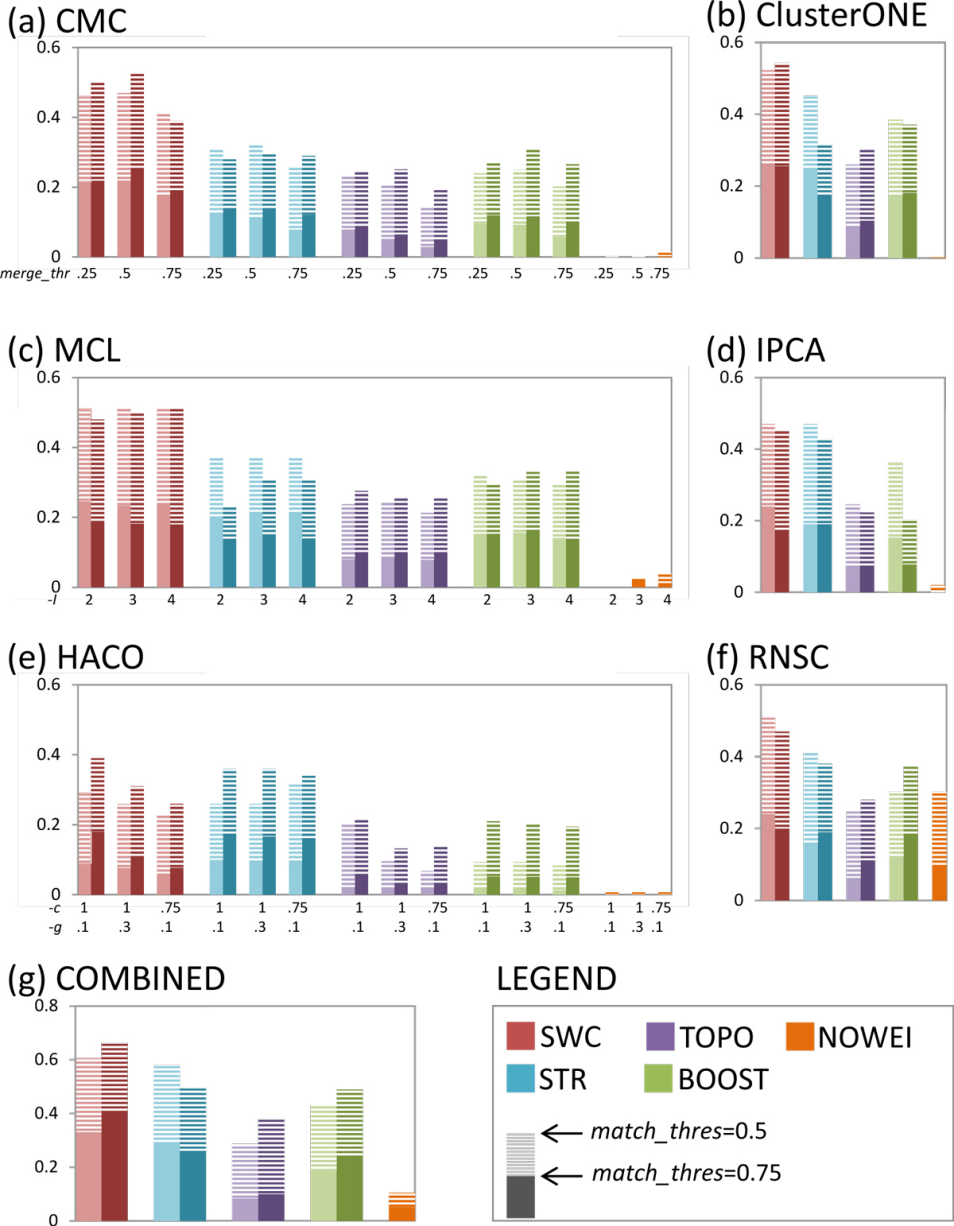
**Precision-recall AUC for yeast complex prediction, using the five weighting approaches for each of the six clustering algorithms and the COMBINED clustering strategy, for *k *= 10000 (lighter shade), *k *= 20000 (darker shade), and *k *= *all edges *(only for NOWEI)**. For CMC, MCL and HACO, three sets of clustering parameters are tried. The AUC for *match_thres *= 0.5 and *match_thres *= 0.75 are shown in each bar. SWC achieves highest precision-recall AUC for all clustering algorithms except IPCA and HACO, where it performs about evenly with STR. The COMBINED strategy achieves higher AUC compared to using any single clustering algorithm alone.

We compare the performance of the five weighting approaches in complex prediction, when each of the six clustering algorithms is used separately, and when all the clustering algorithms are used together with the COMBINED strategy. Figure 3 shows the precision-recall AUC for prediction of yeast complexes, and demonstrates that SWC outperforms the other approaches in most cases: using the best clustering parameter settings for each approach, SWC achieves the highest AUC with all clustering algorithms except for IPCA (where SWC performs about evenly with STRING but outperforms all other approaches) and HACO (where SWC outperforms STRING only for *k *= 20000, but outperforms all other approaches). STRING achieves higher AUC compared to BOOST for all clustering algorithms except for CMC, while BOOST outperforms TOPO for all algorithms except for HACO. Finally, NOWEI performs dismally in all clustering algorithms except for RNSC. The COMBINED strategy achieves higher AUC compared to using each individual clustering algorithm, for all weighting approaches except for NOWEI (its AUC drops compared to using just RNSC alone). Using the COMBINED strategy, SWC achieves the highest AUC, followed by STRING, BOOST, TOPO, and finally NOWEI.

**Figure 4:**
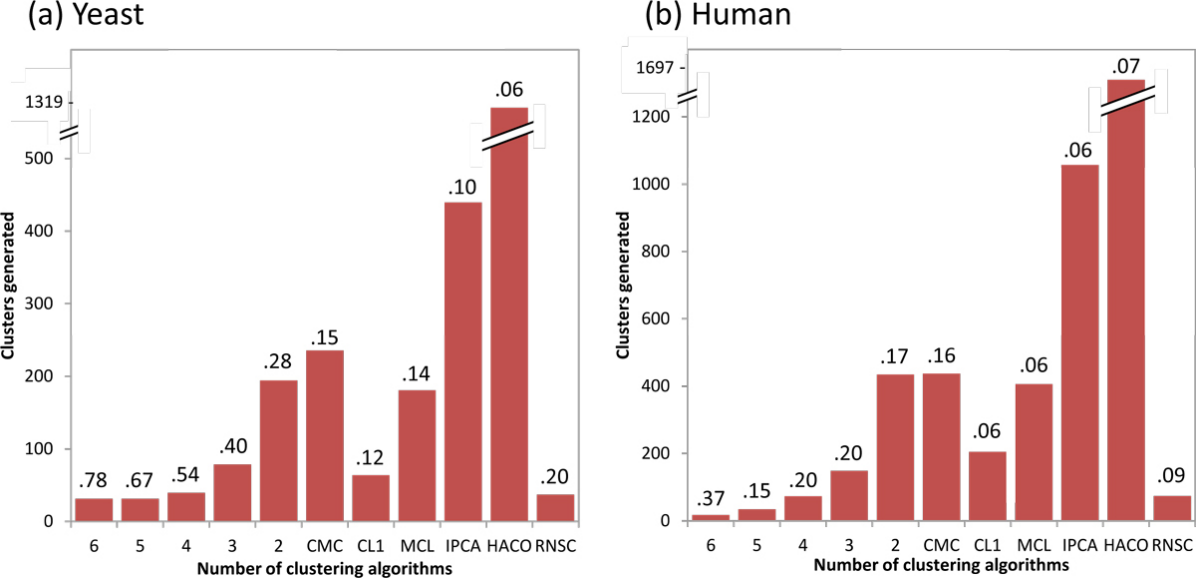
**Distribution of clusters from the COMBINED strategy among any single or multiple number of clustering algorithms that generated them using the SWC network, and their precision (proportion of clusters that match test complexes), in (a) yeast, (b) human**. Different clustering algorithms produce different sets of clusters: in either yeast or human, about 85% of clusters are generated by a single unique algorithm, while less than 7% of clusters are generated by three or more algorithms. Thus aggregating clusters from different algorithms increases the recall of complex prediction. Furthermore, precision increases as clusters are generated by a greater number of algorithms: the highest precision of clusters generated by a single algorithm is 16%, increasing to 78% for clusters generated by all algorithms in yeast, and 37% in human.

We analyze the clusters from the COMBINED strategy to determine how it achieves greater complex-prediction performance by aggregating clusters from the different clustering algorithms with simple voting. Figure 4a shows how clusters from the COMBINED strategy are distributed among any single or multiple number of clustering algorithms that generated them, as well as their precision (the percentage of clusters that match test complexes), in yeast. For brevity we present only the figures for the SWC weighting approach. It reveals that the different algorithms produce different sets of clusters: around 85% of clusters are uniquely generated by a single algorithm, 7% of clusters are generated by two algorithms, and the remaining 8% of clusters are generated by three or more algorithms. Thus, taking their union increases the recall substantially. Furthermore, the precision of clusters increases with the number of algorithms that generated them: among clusters generated by a single algorithm, the highest precision is 20%; clusters generated by two algorithms have a precision of 28%; the precision increases to 78% among the clusters generated by all six algorithms. Thus, voting helps to increase precision by giving greater scores to those clusters predicted by multiple clustering algorithms.

**Figure 5:**
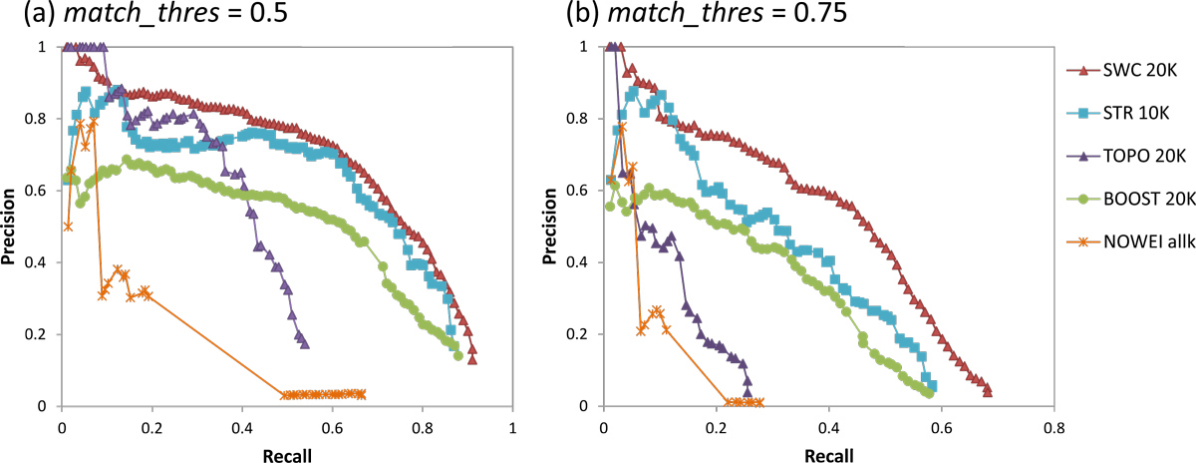
**Precision-recall graphs for yeast complex prediction using the five weighting approaches with the COMBINED clustering strategy, using *k *= 20000 for SWC, TOPO, and BOOST, *k *= 10000 for STR, and *k *= *all edges *for NOWEI**. (a) *match_thres *= 0.5, (b) *match_thres *= 0.75. SWC achieves the highest recall, with the highest precision at almost all recall levels, especially with the stricter *match_thres *= 0.75. Thus it outperforms all other weighting approaches, especially at predicting complexes with fine granularity.

Figure 5 shows the precision-recall graphs for prediction of yeast complexes for the five weighting approaches, using the COMBINED clustering strategy. For brevity, for each approach we show and discuss only the graph for the value of *k *that achieves the highest AUC (*k *= 20000 for SWC, TOPO, and BOOST, *k *= 10000 for STR, *k *= *all *for NOWEI).

SWC recalls the most test complexes, with the highest precision at almost all recall levels, especially with the stricter *match_thres *= 0.75. Thus it outperforms all other weighting approaches, especially at predicting complexes with ne granularity.

At *match_thresh *= 0.5, STR achieves almost the same recall as SWC with only slightly lower precision levels, but its recall and precision are much worse at a higher *match_thresh *= 0.75. Since STR classifies co-complex edges across a large range of clusters, it is able to recall many test complexes; but its lower accuracy in edge classification means that many of its clusters include extra or missing proteins, causing them not to be matched at a stricter matching threshold. BOOST achieves similar recall as STR but with substantially lower precision levels at both match thresholds. Since it classifies edges categorically, many edges have similar scores that do not vary with classification accuracy; thus the ranking of clusters (based on their weighted-densities) does not correlate as well with their correctness, giving lower precision levels. TOPO achieves the lowest recall of all approaches. While its precision for its highest-scoring clusters is comparable to SWC's at *match_thresh *= 0.5 (at the extreme left end of the graph), it drops rapidly for the remaining clusters. This is because TOPO classifies co-complex edges accurately for a limited number of complexes which are thus easy to predict, while the remaining complexes' edges are not as accurately classified, creating many false positive clusters and low recall. Finally, although NOWEI achieves slightly higher recall than TOPO, it generates a great number of false positives, giving extremely low precision.

#### Human

**Figure 6:**
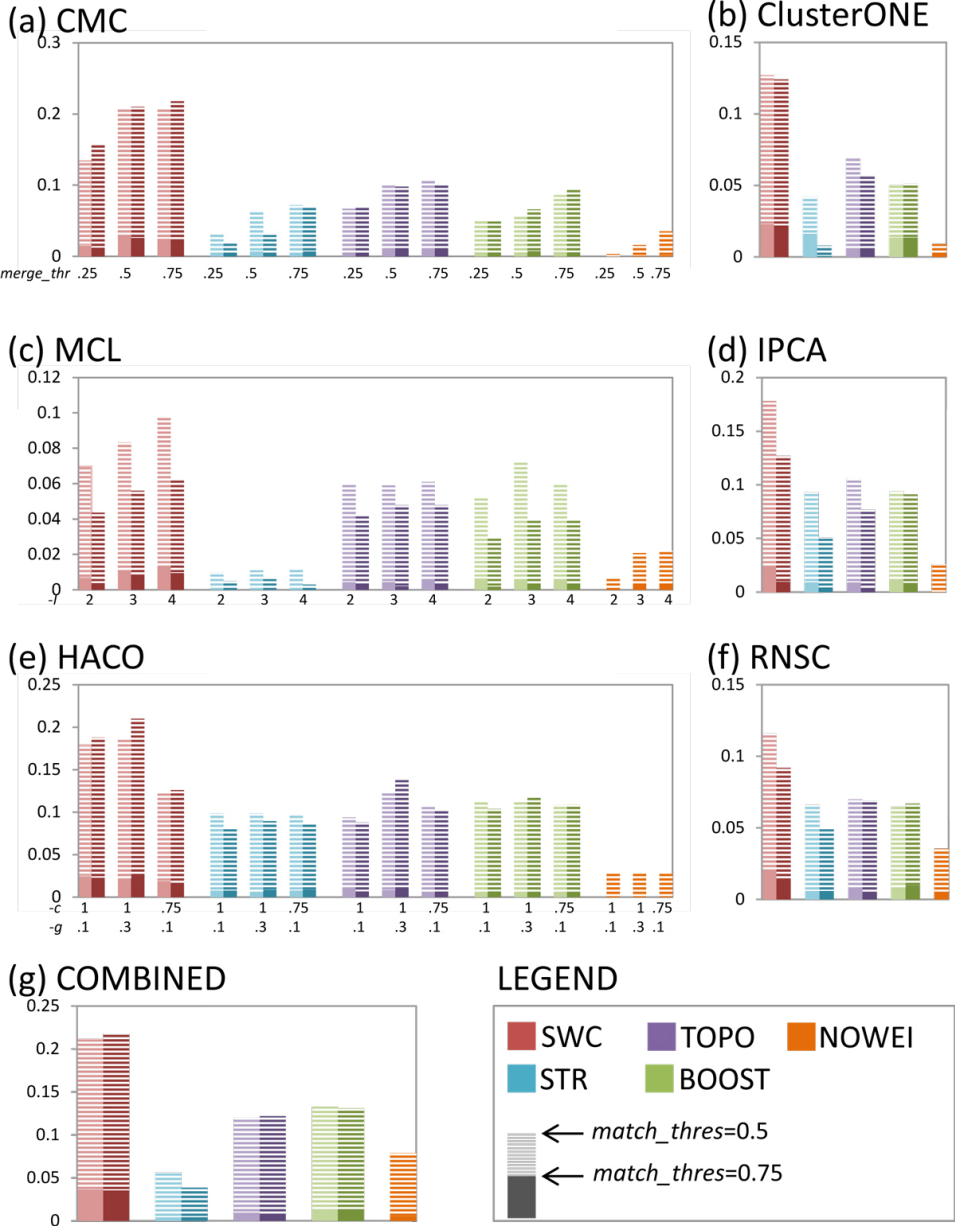
**Precision-recall AUC for human complex prediction, using the five weighting approaches for each of the six clustering algorithms and the COMBINED clustering strategy, for *k *= 10000 (lighter shade), *k *= 20000 (darker shade), and *k *= *all edges *(only for NOWEI)**. For CMC, MCL and HACO, three sets of clustering parameters are tried. The AUC for *match_thres *= 0.5 and *match_thres *= 0.75 are shown in each bar. SWC consistently achieves highest precision-recall AUC for all clustering algorithms and the COMBINED strategy. The COMBINED strategy achieves higher AUC compared to using any single clustering algorithm alone.

Figure 6 shows the precision-recall AUC of the five weighting approaches for the prediction of human complexes. The AUC here is considerably lower than for prediction of yeast complexes, especially at *match_thresh *= 0.75. Nevertheless, it is clear that SWC outperforms all the other weighting approaches. Using each clustering algorithm's best parameter settings for each approach, SWC achieves substantially higher AUC than all the other approaches, for all clustering algorithms. TOPO has higher or similar AUC compared to BOOST for all clustering algorithms except MCL, while STRING performs worse than BOOST for most clustering algorithms except IPCA and RNSC, where their performances are about even. The COMBINED strategy shows less clear benefits for human complexes, in terms of AUC: it gives an improvement for SWC only for *match_thresh *= 0.75, and actually gives worse performance for STRING compared to using CMC, IPCA, RNSC, or HACO alone. Figure 4b shows the distribution of clusters from the COMBINED strategy for SWC in human. As in yeast, around 85% of clusters are uniquely generated by any single clustering algorithm. The precision of the clusters increases as they are generated by more clustering algorithms: from a maximum of 16% when generated by a single algorithm, to 37% when generated by all six algorithms.

**Figure 7:**
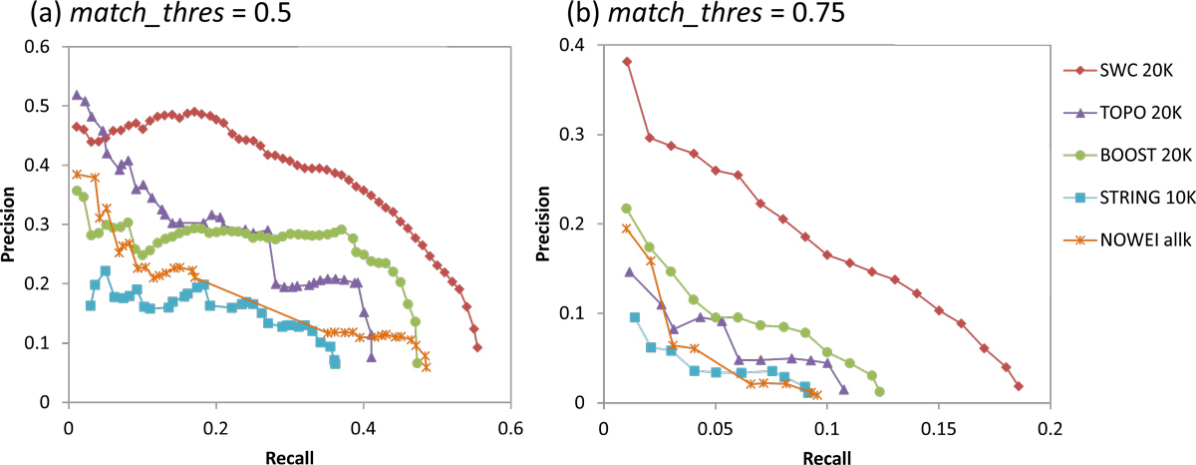
**Precision-recall graphs for human complex prediction using the five weighting approaches for the COMBINED clustering strategy**. SWC achieves the highest recall with the highest precision at almost all recall levels, especially with the stricter *match_thres *= 0.75, where SWC recalls at least 50% more test complexes compared to the other approaches and maintains almost twice the precision throughout its recall range. Thus it outperforms all other weighting approaches, especially at predicting complexes with ne granularity.

Figure 7 shows the precision-recall graphs for prediction of human complexes for the five weighting approaches, using the COMBINED clustering strategy. For brevity, for each approach we show and discuss only the graph for the value of *k *that achieves the highest AUC (*k *= 20000 for SWC, TOPO, and BOOST, *k *= 10000 for STR, *k *= *all *for NOWEI).

SWC attains the highest recall at both *match_thresh*, with higher precision at all recall levels (except that TOPO's top-scoring clusters has slightly higher precision at *match_thresh *= 0.5). The performance advantage is even more pronounced at *match_thresh *= 0.75, where SWC recalls 50% more test complexes compared to the other approaches, and maintains almost twice the precision throughout its recall range. BOOST attains the next highest recall, but with substantially lower precision at all recall levels. Just as in yeast, its categorical edge classification reduces the correctness of the ranking of its clusters, giving lower precision levels.

TOPO achieves lower recall, but at *match_thresh *= 0.5 its precision for its high-scoring clusters is higher than that of BOOST, and even comparable to SWC's for its highest-scoring clusters. Once again, TOPO's high accuracy in classifying edges for a limited number of complexes means it is only able to predict a few complexes well at rough granularity.

Unlike in yeast, here STR performs extremely poorly with the lowest recall and precision levels of all weighting approaches. This is not surprising given that STR performs poorly in edge classification as well. Indeed, even NOWEI achieves higher recall and precision at *match_thresh *= 0.5, with a similar recall at the higher match threshold.

### Prediction of novel complexes

We evaluate the five weighting approaches (SWC, STRING, TOPO, BOOST, and NOWEI) on the number and quality of high-confidence novel complexes predicted in yeast and human. For the supervised approaches (SWC and BOOST), we use the entire reference set of complexes (CYC2008 for yeast, CORUM for human) for training. Next, the edges of the entire network are weighted, and the top *k *edges are used to predict complexes with the COMBINED clustering strategy, which combines clusters predicted by the six clustering algorithms. We use *k *= 20000 for SWC, BOOST, and TOPO, *k *= 10000 for STRING, and *k *= *all edges *for NOWEI.

We filter the set of predicted complexes to obtain a set of unique, novel, high-confidence predictions. First, complexes that are too similar are removed: if any two predicted complexes match with *match_thres *= 0.5, then the complex with the lower score is removed. Next, only novel predictions are kept: if any predicted complex matches any reference complex with *match_thres *= 0.5, then that predicted complex is removed. Finally, only high-confidence predictions are kept: for each weighting approach, using the cross-validation results, the score of each predicted complex is benchmarked to a precision value, and predicted complexes whose estimated precision are less than a confidence threshold are removed. For yeast, this confidence threshold is 0.5; for human, since much fewer complexes are predicted with high precision, we use a 0.4 confidence threshold.

#### Yeast

**Figure 8:**
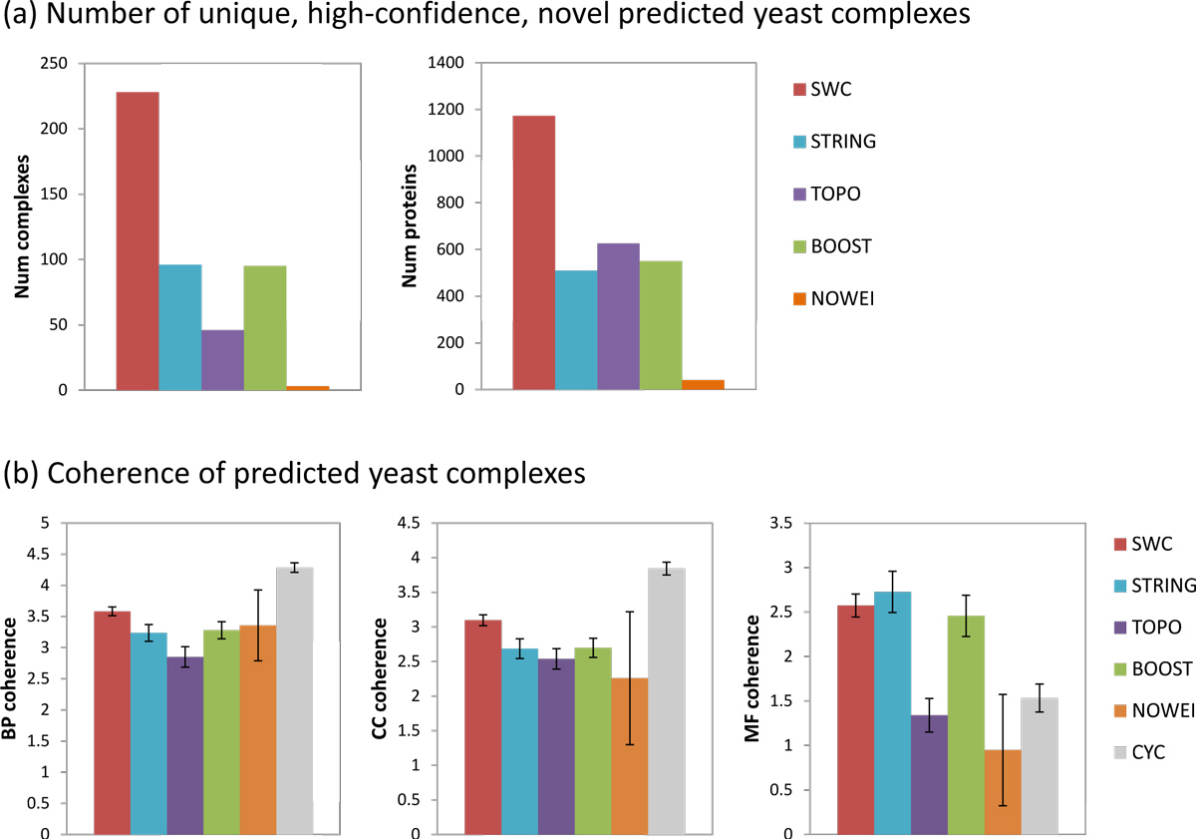
**Unique, high-confidence, novel predicted yeast complexes**. (a) Number of complexes predicted and number of proteins covered. (b) Semantic coherence of predicted complexes. (a) Number of yeast complexes predicted and number of proteins covered, using the five weighting approaches and the COMBINED clustering strategy. SWC generates more novel complexes that cover a greater number of proteins. (b) BP, CC, and MF semantic coherence of the predicted complexes and the reference complexes CYC2008. SWC's complexes have higher BP and CC coherence compared to the other weighting approaches (*p *< 0.05) except for NOWEI (for which there were too few predictions to obtain a significant *p-value*), but lower BP and CC coherence compared to the CYC2008 complexes. The MF coherence for SWC, STRING, BOOST, and CYC2008 complexes are similar, and are higher than that of TOPO or NOWEI (*p *< 0: 0005).

Figure 8a shows the number of novel yeast complexes predicted using the five weighting approaches and the COMBINED clustering strategy. SWC predicts 228 yeast complexes covering 1173 proteins, substantially more than any of the other weighting approaches. Figure 8b shows the BP, CC, and MF coherence of the novel predicted yeast complexes. SWC's complexes have higher BP and CC coherence compared to the other approaches (*p *< 0.05) except for NOWEI (for which there are only three predictions, making it difficult to obtain a significant *p*-*value*); however, the reference complexes of CYC2008 still have much higher BP and CC coherence (*p *< 0.0005). The MF coherence for SWC, STRING, and BOOST complexes are similar to that of the reference complexes, and are much higher than that of TOPO, NOWEI (*p *< 0.0005). Thus, weighting by SWC generates a larger number of novel yeast complexes with greater BP and CC semantic coherence and similar MF coherence, compared to the other weighting approaches. To explore the functions of the novel predicted complexes, we select a set of eleven high-level BP terms, and annotate a novel complex with a BP if that BP is annotated to the most number and a majority of proteins in the complex. Some complexes may be annotated to more than one high-level term. Table 3 shows that almost half of the predicted novel yeast complexes participate in metabolic processes, while the remainder are involved in regulation, cell organization, transport, cellular response, and cell cycle processes. The list of predicted high-confidence novel yeast complexes is given in Additional file 1.

**Table 3:** High-level biological processes of novel predicted yeast complexes

Biological process	# complexes
Protein metabolic process	49
RNA metabolic process	36
DNA metabolic process	15
Small molecule metabolic process	23
Regulation of metabolic process	11
Regulation of gene expression	8
Organelle organization	40
Transport	43
Response to stress	20
Response to chemical stimulus	7
Cell cycle process	11

#### Human

**Figure 9:**
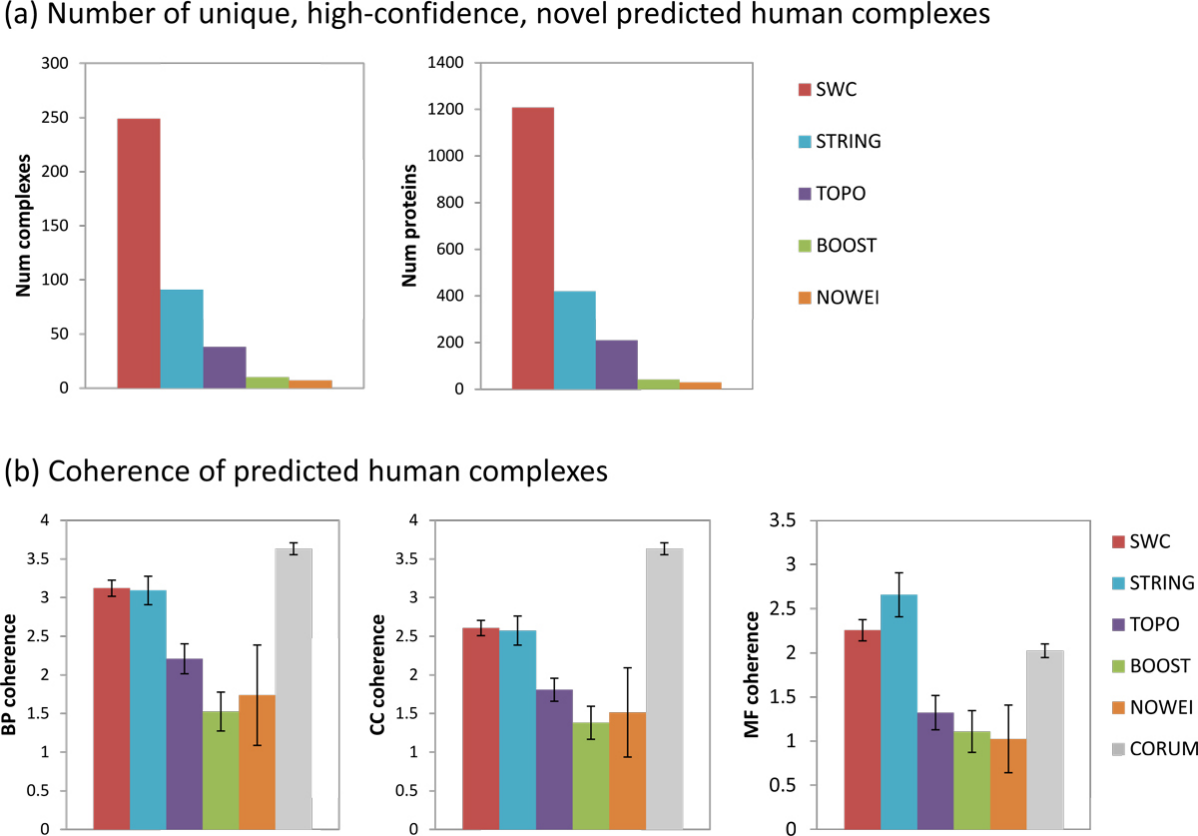
**Unique, high-confidence, novel predicted human complexes**. (a) Number of complexes predicted and number of proteins covered. (b) Semantic coherence of predicted complexes. (a) Number of human complexes predicted and number of proteins covered, using the five weighting approaches and the COMBINED clustering strategy. SWC generates more novel complexes that cover a greater number of proteins. (b) BP, CC, and MF semantic coherence of the predicted complexes and the reference complexes CORUM. SWC's complexes have higher BP, CC, and MF coherence compared to the other weighting approaches (*p *< 0.05 for all, *p *< 0.1 for CC coherence vs NOWEI), but the CORUM complexes have higher BP and CC coherence.

Figure 9 shows the corresponding statistics for the novel predicted human complexes. SWC predicts 249 human complexes covering 1207 proteins, substantially more than any of the other weighting approaches. SWC's complexes have higher BP, CC, and MF coherence compared to those of TOPO (*p *< 0.0005), BOOST (*p *< 0.0005), and NOWEI (*p *< 0.05 for BP and MF, *p *< 0.1 for CC), and have similar BP, CC, and MF coherence compared to those of STR. However, the reference complexes of Corum still have significantly higher BP and CC coherence (*p *< 0.0005), while the MF coherence of SWC and STR are significantly higher than that of the reference complexes (*p *< 0.05). Thus, weighting by SWC generates a larger number of novel human complexes, with greater semantic coherence than other weighting approaches except for STR.

Table 4 shows how many of the predicted human complexes participate in eleven high-level BP terms. About a quarter of the predicted complexes participate in metabolic processes, another quarter in regulation, and the remainder in cell organization, transport, cellular response, and cell cycle processes. The list of predicted high-confidence novel human complexes is given in Additional file 2.

**Table 4:** High-level biological processes of novel predicted human complexes

Biological process	# complexes
Protein metabolic process	32
RNA metabolic process	29
DNA metabolic process	4
Small molecule metabolic process	19
Regulation of metabolic process	74
Regulation of gene expression	34
Organelle organization	19
Transport	38
Response to stress	28
Response to chemical stimulus	32
Cell cycle process	14

### Analysis of learned parameters

**Figure 10:**
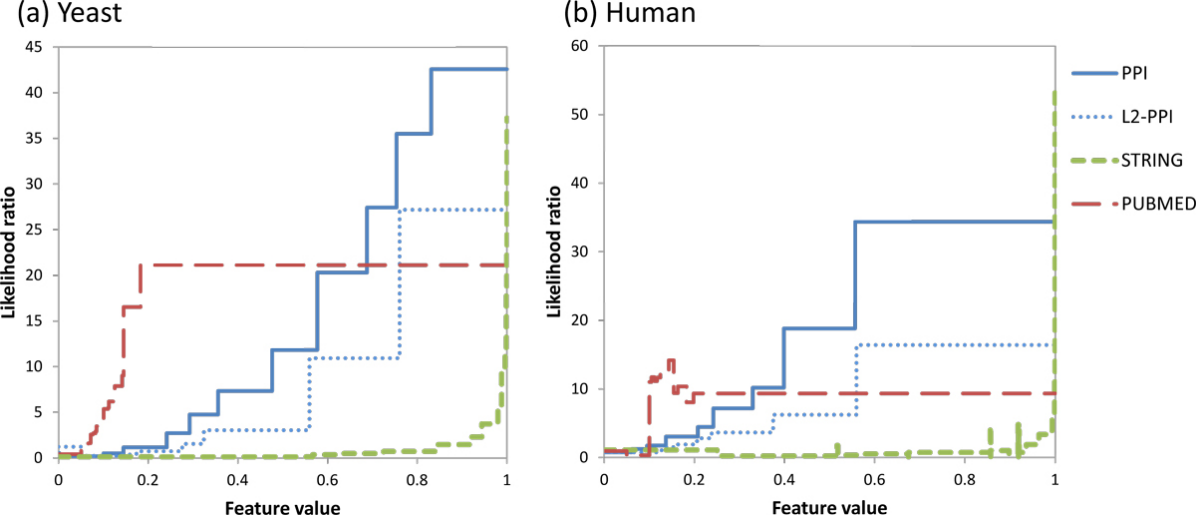
**Learned likelihood parameters, expressed as likelihood ratios, for (a) yeast, (b) human**. For PPI data, interacting proteins are likelier to be co-complex when they have more shared neighbors. For L2-PPI data, where protein pairs are not reported to interact yet share many interaction partners, proteins are also likelier to be co-complex when they have more shared neighbors, but at lower likelihood ratios than corresponding PPI values. For STRING data, protein pairs with predicted functional associations are very likely to be co-complex when the prediction score is high; at low scores, protein pairs are not much likelier to be co-complex. For PubMed data, protein pairs that co-occur in literature, even infrequently, are already much likelier to be co-complex; however, pairs that co-occur more frequently in literature are not any more likelier to be co-complex compared to pairs that co-occur less frequently.

Figures 10a and 10b show the learned likelihood parameters for yeast and human respectively, when the entire reference sets of complexes (CYC2008 for yeast, CORUM for human) are used for training. The likelihood parameters are expressed as likelihood ratios, or how many times likelier is an edge co-complex rather than not co-complex, given the feature value:

$$likelihood\;ratio=\frac{P\left(F=f|co-complex\right)}{P\left(F=f|non-co-complex\right)}$$

The likelihood ratio is a reflection of "co-complexness strength". In general, the likelihood ratios increase as the scores for the data sources (i.e. the x-axes) increase. For the PPI and L2-PPI data sources, protein pairs with higher scores have greater number of shared neighbors, and are likelier to be co-complex: when the score of PPI is close to 1, indicating that almost all of the protein pair's neighbors are shared, the pair is 40 times likelier to be co-complex in yeast and 35 times likelier to be co-complex in human. L2-PPI scores are imputed in edges whose proteins do not actually interact according to PPI databases, yet who share many interaction partners. These scores have corresponding lower likelihood ratios compared to PPI scores: with a score close to 1, the pair is less than 30 times likelier to be co-complex in yeast and less than 20 times likelier to be co-complex in human.

For the STRING data source, only protein pairs with very high functional association scores are likelier to be co-complex: those with the highest scores are almost 40 times likelier to be co-complex in yeast and 50 times likelier to be co-complex in human, whereas protein pairs with lower functional association scores do not seem any likelier to be co-complex.

For PubMed data, protein pairs that co-occur in literature, even infrequently, are already much likelier to be co-complex: about 20 times likelier in yeast and 10 times likelier in human. However, pairs that co-occur more frequently in literature are not any more likelier to be co-complex compared to pairs that co-occur less frequently.

The likelihood ratios for the different data sources show that the co-complexness strength of each data source does not increase linearly with its score. Moreover, between the different data sources, the relationships between data score and co-complexness are different. Thus, combining data scores across different data sources without factoring their dissimilar co-complexness relationships is evidently unsound, while our supervised approach scales the heterogeneous scores to a uniform co-complexness score in terms of likelihoods, which can then be combined probabilistically using the naive-Bayes formulation.

The high likelihood ratios for the data sources also demonstrate that they are indeed indicative of edges belonging to complexes: during cross-validation for both yeast and human, none of the data sources were removed by feature selection in any round.

### Visualization of example complexes

#### Yeast cytochrome bc1 complex

**Figure 11:**
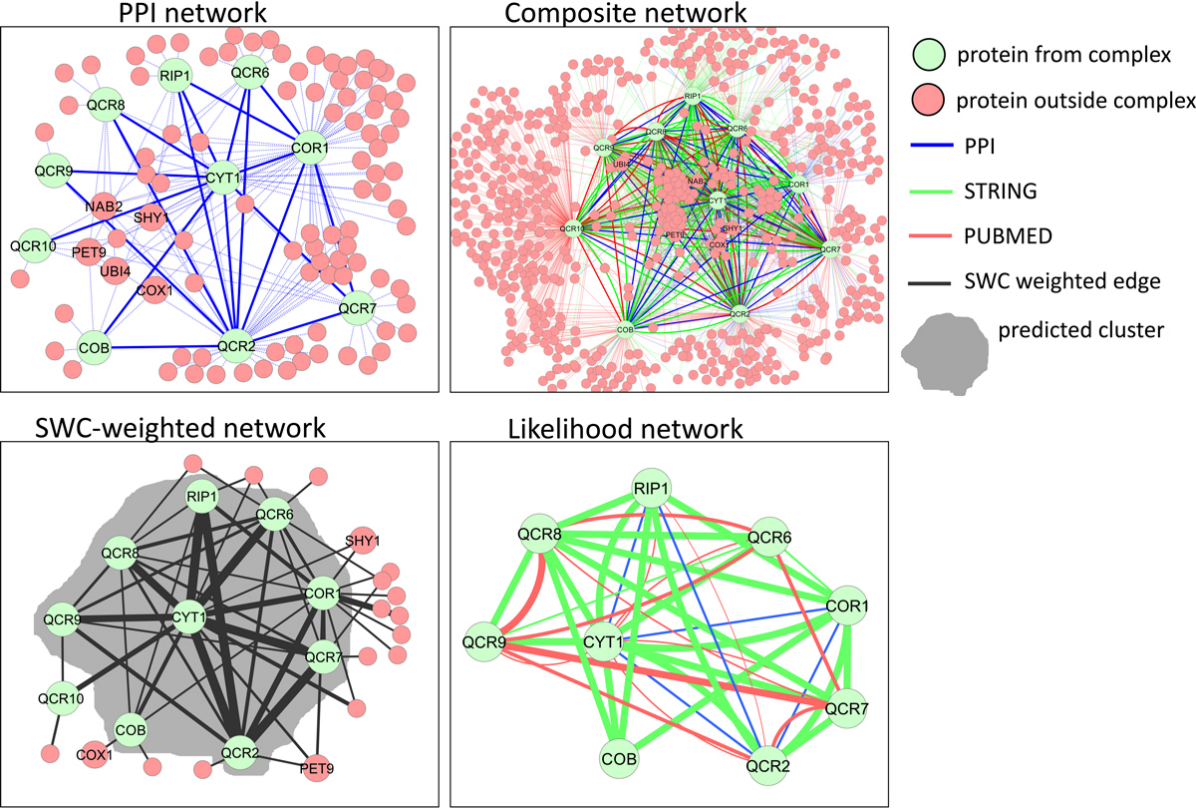
**Yeast mitochondrial cytochrome bc1 complex: PPI network, composite network, SWC-weighted network, and likelihood network**. The PPI subgraph includes many extraneous edges and external neighboring proteins. In the composite network, the extra edges from the other data sources induce a full clique among the complex proteins, although the number of extraneous edges increases dramatically as well. In the SWC-weighted network, the complex's subgraph is still relatively dense, with fewer extraneous edges remaining, allowing the complex to be easily found by both IPCA and RNSC (although missing one protein). In the likelihood network, diverse data sources connect many proteins within the cluster with high SWC scores. CYT1-RIP1-QCR2 are fully connected with each other via all three data sources with moderate to high co-complexness, making them a central triplet within the cluster, while CYT1-COR1-QCR2 and CYT1-QCR7-QCR2 are connected with two or more data sources with moderate to high co-complexness, deeply embedded in the cluster as well. The other proteins appear less central in the cluster, especially COB, a fringe member which is only connected via functional associations to four proteins.

In this section we use two example complexes to illustrate the power and mechanism of SWC. Figure 11a shows the PPI subgraph of the yeast mitochondrial cytochrome bc1 complex discussed earlier, which is involved in the electron-transport chain in the mitochondrial inner membrane. The complex's PPI subgraph has 19 co-complex interactions, and 145 extraneous interactions with 94 external proteins, among which five are labeled: NAB2 and UBI4 are involved in mRNA polyadenylation and protein ubiquitination respectively, and bind to many proteins to perform their functions; PET9, SHY1, and COX1 are mitochondrial membrane proteins that are also involved in the electron-transport chain, and interact with proteins of the complex, although they are not part of it. In the composite network (Figure 11b), the edges from the other data sources induce a full clique among the complex proteins, although the number of extraneous edges and number of neighbors outside the complex increase to 1735 and 640 respectively. After weighting by SWC and selecting the top *k *= 20000 edges (Figure 11c), the complex's subgraph is still relatively dense; furthermore, only 26 extraneous edges and 18 neighboring proteins remain. Note that among the five labeled external proteins, the two involved in unrelated processes (NAB2 and UBI4) have been disconnected at this point, while the three also involved in the electron transport chain with the complex (PET9, SHY1, and COX1) are still connected to the network. With this network, both IPCA and RNSC detect the cluster shaded in gray, which matches the complex with Jaccard similarity of 0.9.

The likelihood network for the cluster (Figure 11d) visualizes the component evidences for the prediction: the contribution of each data source to an edge's SWC score is reflected in the edge thickness, which is scaled with its likelihood ratio, or co-complexness strength. The likelihood network reveals that diverse data sources connect many proteins within the cluster with high SWC scores. CYT1, RIP1, and QCR2 are fully connected with each other via all three data sources, making them the strongest co-complex triplet that is centrally embedded in the cluster, while CYT1-COR1-QCR2 and CYT1-QCR7-QCR2 are connected with two or more data sources, making them highly co-complex and deeply embedded as well. The other proteins appear less central in the cluster, especially COB, a fringe member which is only connected via functional associations to four proteins.

#### Human BRCA1-A complex

**Figure 12:**
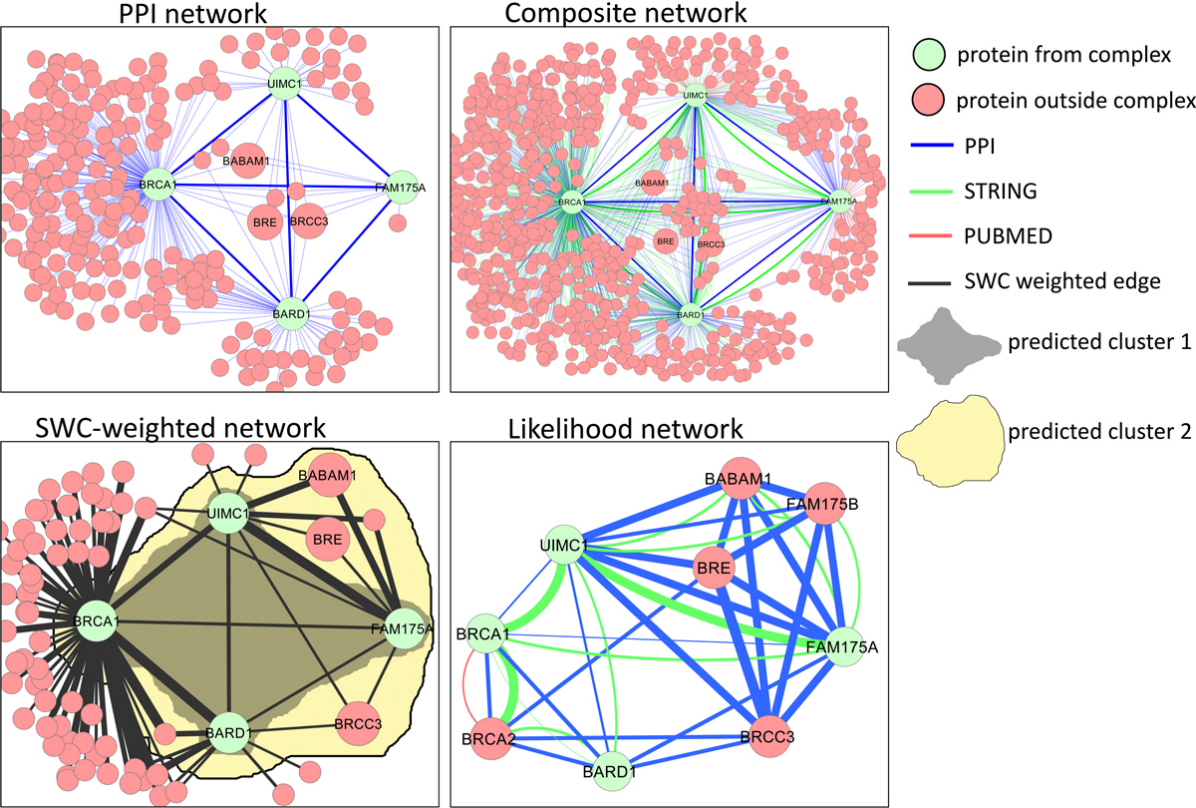
**Human BRCA1-A complex: PPI network, composite network, SWC-weighted network, and likelihood network**. While the PPI network is fully connected, there are large numbers of extraneous edges and neighboring proteins (chiefly because BRCA1 itself is connected to around 180 proteins). In the SWC network, BRCA1 is still connected to a large number of proteins, but most of them are not connected to the other proteins in the complex, so they are unlikely to be clustered together. Clustering this network produces both the cluster consisting of the four complex proteins (generated by CMC), as well as a larger cluster consisting of the four complex proteins plus five additional proteins BABAM1, BRE, BRCC3, BRCA2, and FAM175B. Recent papers indicate that the former three additional proteins have been included in the BRCA1-A complex. The likelihood network shows that the three additional members are completely connected in a clique with two of the original complex members FAM175A and UIMC1 via PPI edges with strong co-complexness, while the four original members themselves are less strongly connected, via two functional associations with high co-complexness and a few low co-complexness PPIs.

Figure 12 shows the human BRCA1-A complex, which is involved in DNA repair. The CORUM reference set of complexes specify that complex consists of four proteins, BRCA1, BARD1, FAM175A, and UIMC1, while a survey of current literature reveals that it is composed of at least three more proteins, BRE, BABAM1, and BRCC3. While the PPI network for this complex is fully connected, there are extremely large numbers of extraneous edges and neighboring proteins, chiefly because BRCA1 itself is connected to around 180 proteins. Note that the three new members BRE, BABAM1, and BRCC3 are also connected to the original complex proteins. After weighting the composite network and keeping the top *k *= 20000 edges, BRCA1 is still connected to a large number of proteins (62), but the majority of them are not connected to the other proteins in the complex, so they are unlikely to be clustered together. Moreover, BRE, BABAM1, and BRCC3 are still highly connected to the original complex proteins. Indeed, clustering this network produces both the cluster consisting of the four CORUM proteins (generated by CMC), as well as a larger cluster consisting of the four CORUM proteins plus the three new members and two extra proteins (generated by IPCA). The likelihood network shows that PPI edges with strong co-complexness induce a full clique between two CORUM complex members FAM175A and UIMC1 with the three new members and an additional protein FAM175B; on the other hand, the four CORUM complex proteins themselves are less strongly connected, via two functional associations with high co-complexness and a few low co-complexness PPIs. This provides ample evidence that the three new proteins belong to this complex, while the inclusion of two extra proteins BRCA2 and FAM175B is likely due to their participation in other complexes that overlap with the BRCA1-A complex.

### Two novel predicted complexes

We select two novel complexes predicted with the COMBINED strategy using the SWC network, with the entire reference set of complexes for training.

**Figure 13:**
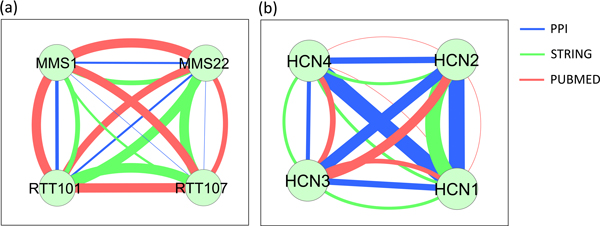
**Two novel predicted complexes**. (a) Novel yeast predicted complex, annotated with DNA metabolic process and response to stress. The four proteins are fully connected by six literature co-occurrences with strong co-complexness, six functional associations with strong or moderate co-complexness, and five PPI edges with moderate or weak co-complexness. The diverse mix of data sources provides convincing evidence for this complex. A scan through the literature reveals that these four proteins form a complex named Cul8-RING ubiquitin ligase complex [28], thought to be involved in DNA repair and regulation of chromatin metabolism, although our set of reference complexes has not been updated to include this complex. (b) Novel human predicted complex annotated with transport process. These proteins are fully connected by six PPIs with strong co-complexness, five functional associations with strong to moderate co-complexness, and five literature co-occurrences with strong to weak co-complexness. The strong PPIs, reinforced by the other data sources, provide high credibility to this prediction. The Uniprot descriptions for these proteins suggest that they may constitute subunits of a potassium channel complex [29].

One high-scoring novel yeast complex, generated by all six clustering algorithms, is composed of four proteins, MMS1, MMS22, RTT101, and RTT107, and is annotated with two high-level BP terms, DNA metabolic process and response to stress. Figure 13a shows its likelihood network. The four proteins are fully connected by six literature co-occurrences with strong co-complexness, and six functional associations with strong or moderate co-complexness. Five PPI edges with moderate or weak co-complexness also connect the proteins. The diverse mix of data sources provides convincing evidence for this complex. A scan through the literature reveals that these four proteins form a complex named Cul8-RING ubiquitin ligase complex [28], thought to be involved in DNA repair and regulation of chromatin metabolism, which the yeast reference complexes set CYC2008 has apparently failed to include.

Figure 13b shows a high-scoring novel human complex, generated by all six clustering algorithms, made up of four proteins, HCN1, HCN2, HCN3, and HCN4, and annotated with one high-level BP term, transport. These proteins are fully connected by six PPIs with strong co-complexness, while five functional associations with strong to moderate co-complexness and five literature co-occurrences with strong to weak co-complexness also connect the proteins. The strong PPIs, reinforced by the other data sources, provide high credibility to this prediction. Indeed, the Uniprot descriptions for these proteins suggest that they may constitute subunits of a potassium channel complex [29].

## Conclusion

In this paper, we introduce a maximum-likelihood supervised approach for weighting composite protein networks for predicting protein complexes, called SWC (Supervised Weighting of Composite networks). First, we construct a composite protein network using three heterogeneous data sources: PPI, predicted functional association, and co-occurrence in literature abstracts. Next, we weight each edge of the composite network based on its posterior probability of belonging to a protein complex, using a naive-Bayes maximum-likelihood model learned from a set of training complexes. The weighted composite network is then used by clustering algorithms to predict new complexes. We also propose a simple aggregative clustering strategy that combines clusters generated by multiple clustering algorithms, using simple voting. We evaluate our weighting scheme using six clustering algorithms, as well our aggregative clustering strategy, on the prediction of yeast and human complexes. We demonstrate that our proposed method outperforms a supervised data-integration approach using boosting, a predicted functional-association network from STRING, an unsupervised approach using a topological function to weight PPI networks, as well as a baseline approach using unweighted PPI networks: our approach predicts more correct complexes at higher precision levels, and generates more high-confidence novel complexes with similar or better semantic coherence. Using a few example complexes, we show that our approach increases the density of the complexes' subgraphs, and filters them to remove extraneous edges. Furthermore, our approach allows visualization of the evidence of predicted complexes, using learned likelihood parameters to express strengths of co-complex relationships of each data type. This aids human evaluation of the credibility of predicted complexes.

Finally, we present two novel predicted complexes: a four-protein yeast complex possibly involved in DNA metabolism and stress response, and a four-protein human complex possibly involved in transport processes. We show that these predictions appear credible from their evidences, being supported by diverse data sources with strong co-complexness. Indeed, a recent paper presents the predicted yeast complex as the Cul8-RING ubiquitin ligase complex, while the Uniprot database provides evidence that the predicted human complex may exist as a potassium channel complex.

SWC software package and data files are available at http://compbio.ddns.comp.nus.edu.sg/~cherny/SWC/.

## Competing interests

The authors declare that they have no competing interests.

## Authors' contributions

CHY derived and implemented the algorithms, performed the experiments, and drafted the manuscript. GL and CHY designed the experiments. HNC and CHY selected and prepared the data sources. LW conceived and directed the study. All authors read and approved the final manuscript.

## Supplementary Material

Additional file 1 **Novel, unique, high-confidence predicted yeast complexes**. Click here for file

Additional file 2 **Novel, unique, high-confidence predicted human complexes**. Click here for file
