# Supplementary material for: Supervised maximum-likelihood weighting of composite protein networks for complex prediction
Source: BMC Syst Biol. 2012 Dec 12;6(Suppl 2):S13. doi: 10.1186/1752-0509-6-S2-S13 (PMC3521185; doi:10.1186/1752-0509-6-S2-S13)
Supplement: Additional file 1 — Novel, unique, high-confidence predicted yeast complexes. [file 1752-0509-6-S2-S13-S1.pdf]

>CMCL_C34|6|CL1|CMC|HACO|IPCA|MCL|RNSC| 11 5.41003705348435 cell cycle process
YBR107C
YBR211C
YDR254W
YDR318W
YDR383C
YGR179C
YJR135C
YLR315W
YLR381W
YPL018W
YPR046W
>CHACO_C6761|6|CL1|CMC|HACO|IPCA|MCL|RNSC| 4 5.30915167797728 DNA metabolic process, response to stress
YHR154W
YJL047C
YLR320W
YPR164W
>CIPCA_C510|6|CL1|CMC|HACO|IPCA|MCL|RNSC| 4 5.25616620046669 small molecule metabolic process
YCR048W
YNR008W
YNR019W
YOR245C
>CCMC_C26|6|CL1|CMC|HACO|IPCA|MCL|RNSC| 4 5.10240950646192 small molecule metabolic process, response to stress
YLR155C
YLR157C
YLR158C
YLR160C
>CMCL_C156|6|CL1|CMC|HACO|IPCA|MCL|RNSC| 5 4.68073270874892 others

YBL068W
YER099C
YHL011C
YKL181W
YOL061W

>CCMC_C125|6|CL1|CMC|HACO|IPCA|MCL|RNSC| 4 4.42389162479702 small molecule metabolic process
YAR073W
YHR216W
YLR432W
YML056C
>CRNSC_C184|6|CL1|CMC|HACO|IPCA|MCL|RNSC| 4 4.25227656556776 small molecule metabolic process
YKL088W
YKR072C
YML016C
YOR054C
>CRNSC_C70|6|CL1|CMC|HACO|IPCA|MCL|RNSC| 9 4.17498933034752 transport
YCL011C
YDL084W
YDR138W
YDR381W
YHR167W
YML062C
YNL004W
YNL139C
YNL253W
>CCMC_C231|6|CL1|CMC|HACO|IPCA|MCL|RNSC| 6 3.89806416770964 small molecule metabolic process
YFL059W
YFL060C
YMR095C
YMR096W
YNL333W
YNL334C
>CCMC_C53|5|CL1|CMC|IPCA|MCL|RNSC|8 3.75665895077169 transport
YDL194W
YDR342C
YDR343C
YDR345C
YHR092C
YHR094C

YHR096C
YMR011W
>CIPCA_C118|5|CL1|CMC|IPCA|MCL|RNSC| 14 3.6084704335918 small molecule metabolic process
YER044C
YGL001C
YGL012W
YGR060W
YGR175C
YHR007C
YHR072W
YHR190W
YLR056W
YLR100W
YML008C
YMR015C
YMR202W
YNL280C
>CHACO_C7701|4|CMC|HACO|IPCA|RNSC| 21 3.47541684541296 protein metabolic process, organelle organization
YBL038W
YBR122C
YCR046C
YCR071C
YDR116C
YDR237W
YDR296W
YDR322W
YDR462W
YGR220C
YJL063C
YKR006C
YKR085C
YLR312W-A
YLR439W
YML025C
YMR024W
YMR193W

YNL005C
YNL252C
YOR150W
>CRNSC_C186|6|CL1|CMC|HACO|IPCA|MCL|RNSC| 8 3.46827901697942
transport
YBL078C
YBR217W
YHR171W
YLL042C
YMR159C
YNL223W
YNR007C
YPL149W
>CCMC_C271|6|CL1|CMC|HACO|IPCA|MCL|RNSC| 4 3.33950352676893
protein metabolic process, response to stress
YDL079C
YMR139W
YNL307C
YOL128C
>CHACO_C7562|5|CL1|CMC|HACO|IPCA|RNSC| 6 3.1585278931646
protein metabolic process
YDR214W
YKL117W
YLR216C
YMR186W
YOR027W
YPL240C
>CHACO_C9071|5|CL1|CMC|HACO|IPCA|RNSC| 4 3.1582524699293 others

YBR018C
YBR019C
YBR020W
YLR081W
>CCMC_C191|5|CL1|CMC|IPCA|MCL|RNSC| 7 3.03826583988376 others
YCR107W
YDL243C
YFL056C
YFL057C

YJR155W
YNL331C
YOL165C
>CCMC_C161|4|CL1|CMC|HACO|RNSC| 5 2.87106164952969 transport, organelle organization
YDR468C
YGL095C
YMR197C
YOL018C
YOR036W
>CCMC_C180|4|CL1|CMC|HACO|RNSC| 5 2.86483126411356 protein metabolic process, organelle organization
YBL105C
YHR030C
YJL095W
YOR231W
YPL140C
>CHACO_C9106|4|CMC|HACO|MCL|RNSC| 5 2.85352521011432 regulation of gene expression, regulation of metabolic process
YGR112W
YIL157C
YJL062W-A
YLR203C
YML129C
>CRNSC_C82|4|CL1|IPCA|MCL|RNSC| 4 2.60879422472599 protein metabolic process
YDR490C
YKL126W
YMR104C
YOL100W
>CHACO_C7381|3|CMC|HACO|RNSC| 11 2.55359680861281 others
YDR244W
YDR265W
YGL153W
YGR077C
YJL210W
YKL197C
YLR191W

YMR026C
YNL214W
YNL329C
YOL044W
>CCMC_C216|4|CL1|CMC|IPCA|RNSC| 5 2.50390649865546 small molecule metabolic process
YFR030W
YJR010W
YJR137C
YKL001C
YPR167C
>CHACO_C6896|3|CMC|HACO|MCL| 4 2.49474574506521 protein metabolic process
YDR422C
YER027C
YGL115W
YGL208W
>CRNSC_C10|3|CMC|IPCA|RNSC| 4 2.37944357369337 others
YGR192C
YJL052W
YJR009C
YKL060C
>CRNSC_C205|4|CL1|CMC|IPCA|RNSC| 4 2.27495708205756 response to chemical stimulus, small molecule metabolic process, response to stress
YGR256W
YHR183W
YNL241C
YPR074C
>CCMC_C129|3|CMC|HACO|IPCA| 4 2.26325729995405 transport
YAL007C
YAR002C-A
YGL200C
YML012W
>CHACO_C7363|3|CMC|HACO|RNSC| 6 2.22573177691594 transport, small molecule metabolic process
YBL099W
YBR039W
YDR298C

YJR121W
YKL016C
YPL078C
>CRNSC_C26|3|HACO|MCL|RNSC| 6 2.17498866061712 RNA metabolic process
YDR021W
YGL078C
YGL171W
YHR065C
YHR169W
YKR024C
>CCMC_C217|4|CMC|IPCA|MCL|RNSC| 5 2.15684535772697 transport
YBR296C
YCR037C
YJL198W
YML123C
YNR013C
>CIPCA_C99|4|CL1|IPCA|MCL|RNSC| 14 2.12221310774477 DNA metabolic process, organelle organization
YBL113C
YDR545W
YEL077C
YER190W
YGR296W
YIL177C
YJL225C
YLL066C
YLL067C
YLR466W
YLR467W
YML133C
YNL339C
YPL283C
>CCMC_C210|4|CMC|IPCA|MCL|RNSC| 7 2.11030680514455 response to stress
YDL235C
YER118C
YIL147C

YJL128C
YLR006C
YLR113W
YNR031C
>CHACO_C7340|3|CMC|HACO|RNSC| 5 2.10423801783997 others
YDL067C
YGL187C
YHR051W
YLR038C
YNL052W
>CCMC_C44|3|CMC|IPCA|RNSC| 42 2.0838830669807 RNA metabolic process
YBL004W
YBR247C
YCL059C
YCR057C
YDL014W
YDL148C
YDR324C
YDR449C
YER082C
YGR090W
YGR128C
YGR145W
YHR148W
YHR196W
YIL019W
YJL010C
YJL069C
YJL109C
YJR002W
YKR060W
YLL011W
YLR129W
YLR186W
YLR197W
YLR222C
YLR409C
YMR093W

YMR128W
YMR229C
YMR290C
YNL075W
YNL110C
YNL132W
YOL010W
YOR078W
YOR145C
YOR310C
YPL012W
YPL126W
YPL217C
YPR137W
YPR144C
>CRNSC_C177|4|CL1|IPCA|MCL|RNSC| 7 2.05627503129385 response to chemical stimulus, transport
YBL005W
YDR011W
YDR406W
YGL013C
YGR281W
YOR153W
YOR328W
>CCL1_C254|4|CL1|IPCA|MCL|RNSC| 4 2.04226212804902 transport
YGL255W
YJL056C
YKL175W
YLR130C
>CRNSC_C55|3|CL1|CMC|RNSC| 5 2.02878434634641 transport, response to stress
YHR114W
YJL020C
YKL129C
YMR109W
YOR181W
>CCMC_C282|4|CMC|HACO|IPCA|RNSC| 4 2.01994369308414 transport
YCR073W-A

YGR248W
YHR163W
YNR034W
>CCMC_C30|3|CMC|IPCA|RNSC| 46 2.01187208785168 RNA metabolic process
YBR142W
YCL054W
YDL031W
YDR060W
YDR087C
YDR101C
YDR496C
YER006W
YER126C
YFL002C
YFR001W
YGL111W
YGR103W
YGR245C
YHR052W
YHR066W
YHR088W
YHR197W
YKL009W
YKL021C
YKL172W
YKR081C
YLL008W
YLL034C
YLR002C
YLR009W
YLR222C
YLR276C
YLR449W
YMR049C
YMR229C
YMR290C
YNL002C
YNL061W

YNL110C
YNR053C
YOL041C
YOL077C
YOR063W
YOR206W
YOR272W
YPL043W
YPL093W
YPL131W
YPL211W
YPR016C
>CCMC_C181|3|CL1|CMC|IPCA| 4 1.99639209193737 RNA metabolic process
YDL175C
YLR430W
YNL251C
YPL190C
>CCMC_C158|3|CMC|IPCA|RNSC| 4 1.9834136988477 organelle organization
YBR179C
YIL065C
YJL112W
YLL001W
>CRNSC_C122|3|CMC|IPCA|RNSC| 33 1.93428595483723 RNA metabolic process
YBR055C
YBR152W
YDL030W
YDL043C
YDL098C
YDR473C
YER029C
YER172C
YFL017W-A
YGR074W
YGR091W
YHR165C
YJL203W

YKL173W
YLL036C
YLR117C
YLR147C
YLR275W
YLR424W
YML046W
YML049C
YMR125W
YMR213W
YMR240C
YMR288W
YOR159C
YOR308C
YPL151C
YPL213W
YPR082C
YPR101W
YPR178W
YPR182W
>CRNSC_C30|4|CMC|HACO|IPCA|RNSC| 4 1.90413896072049 transport
YAL026C
YDR093W
YER166W
YMR162C
>CCMC_C18|3|CMC|IPCA|RNSC| 87 1.89849404954882 protein metabolic process

YBL027W
YBL072C
YBL092W
YBR031W
YBR048W
YBR181C
YBR189W
YBR191W
YCR031C
YDL075W
YDL082W

YDL083C
YDL136W
YDR012W
YDR025W
YDR064W
YDR382W
YDR418W
YDR447C
YDR450W
YDR471W
YER074W
YER102W
YFR031C-A
YGL030W
YGL031C
YGL076C
YGL103W
YGL135W
YGL147C
YGR034W
YGR085C
YGR118W
YGR214W
YHL001W
YHL033C
YHR141C
YHR203C
YIL018W
YIL069C
YIL133C
YJL177W
YJL190C
YJL191W
YJR123W
YJR145C
YKL180W
YKR094C
YLL045C

YLR029C
YLR048W
YLR075W
YLR287C-A
YLR333C
YLR340W
YLR344W
YLR388W
YLR441C
YLR448W
YML063W
YML073C
YMR142C
YMR194W
YMR242C
YNL067W
YNL069C
YNL096C
YNL110C
YNL162W
YNL178W
YNL301C
YNL302C
YOL040C
YOL120C
YOL127W
YOR063W
YOR096W
YOR234C
YOR293W
YOR312C
YPL090C
YPL131W
YPL143W
YPL198W
YPL220W
YPL249C-A
YPR102C

>CCMC_C107|3|CL1|CMC|IPCA| 6 1.86890789949124 cell cycle process, DNA metabolic process, response to stress
YCR092C
YDR097C
YMR167W
YNL082W
YOL090W
YPL164C
>CRNSC_C152|3|CMC|IPCA|RNSC| 9 1.86764182156646 others
YAL024C
YAR019C
YFR028C
YGR092W
YHR152W
YJR053W
YML064C
YMR001C
YMR055C
>CCMC_C313|6|CL1|CMC|HACO|IPCA|MCL|RNSC| 4 1.8509294429409 small molecule metabolic process
YDR007W
YEL021W
YOR128C
YOR202W
>CHACO_C5760|2|CMC|HACO| 4 1.82436200835846 RNA metabolic process
YDL208W
YHR072W-A
YHR089C
YLR175W
>CHACO_C7565|3|CMC|HACO|RNSC| 5 1.79643478183965 small molecule metabolic process
YDR148C
YGR244C
YIL125W
YOR142W
YPL262W
>CRNSC_C75|3|CMC|HACO|RNSC| 4 1.77082393598662 DNA metabolic process

YAL029C
YBL031W
YBR130C
YKL130C
>CCL1_C103|3|CL1|MCL|RNSC| 6 1.76544209225908 cell cycle process, regulation of gene expression, regulation of metabolic process
YDR146C
YIL131C
YLR131C
YMR043W
YNL068C
YOR372C
>CCMC_C259|3|CMC|IPCA|RNSC| 5 1.76331264044646 protein metabolic process, organelle organization
YBR114W
YEL037C
YER162C
YJR052W
YMR201C
>CHACO_C7307|2|HACO|RNSC| 6 1.73967587699183 cell cycle process
YCR002C
YDL225W
YHR107C
YJR076C
YLR314C
YNL166C
>CCMC_C189|3|CMC|IPCA|RNSC| 5 1.73500387605466 others
Q0045
Q0085
Q0250
Q0275
YLR203C
>CCMC_C214|3|CMC|IPCA|RNSC| 11 1.72063243496016 regulation of gene expression, regulation of metabolic process
YBR279W
YGL019W
YGL207W
YGL244W

YIL035C
YLR418C
YML069W
YOL145C
YOR039W
YOR061W
YOR123C
>CCMC_C284|4|CL1|CMC|IPCA|RNSC| 4 1.69771437547128 cell cycle process
YER044C-A
YHR157W
YLR329W
YMR133W
>CCL1_C113|4|CL1|IPCA|MCL|RNSC| 7 1.66847362525149 protein metabolic process
YBL082C
YGR227W
YNL219C
YNR030W
YOR002W
YOR067C
YPL227C
>CCMC_C264|3|CMC|HACO|RNSC| 8 1.66669296600036 regulation of metabolic process
YBR160W
YDL155W
YGR108W
YGR109C
YLR079W
YLR210W
YPR119W
YPR120C
>CHACO_C6656|2|CMC|HACO| 4 1.66287647765092 response to chemical stimulus, transport
YDR011W
YGR281W
YOR153W
YPL058C

>CCMC_C71|3|CMC|IPCA|RNSC| 24 1.65747334072059 transport
YBL079W
YDL088C
YDL116W
YDL207W
YDR159W
YDR192C
YER105C
YER107C
YFR002W
YGL092W
YGL100W
YGL172W
YGR119C
YIL115C
YJL061W
YJR042W
YKL057C
YKL068W
YKR082W
YLR018C
YML103C
YMR047C
YMR129W
YPL169C
>CHACO_C6650|2|CMC|HACO| 4 1.65115786373423 transport, small molecule metabolic process
YDR377W
YKL016C
YML081C-A
YPL078C
>CHACO_C7671|3|CL1|CMC|HACO| 4 1.58869184223803 transport
YDL149W
YFR021W
YLR431C
YNL242W
>CCL1_C154|2|CL1|RNSC| 4 1.55751978428953 organelle organization
YCL029C

YER016W
YLR045C
YPL269W
>CHACO_C7660|2|CMC|HACO| 6 1.55459657863522 organelle organization
YBL050W
YGL212W
YKL196C
YLR093C
YMR197C
YOR106W
>CHACO_C7175|2|CMC|HACO| 4 1.53286675098119 response to stress
YBL088C
YBR136W
YML058W
YPL153C
>CCMC_C50|2|CMC|RNSC| 9 1.52148819160635 organelle organization
YBL050W
YDR189W
YHL031C
YIL004C
YKL006C-A
YKL196C
YLR026C
YLR078C
YLR268W
>CHACO_C7099|2|CMC|HACO| 4 1.52122878173036 others
YER165W
YGL049C
YGR162W
YOL139C
>CCMC_C279|3|CMC|HACO|IPCA| 4 1.51802522081184 others
YEL011W
YFR015C
YKR058W
YLR258W
>CIPCA_C470|6|CL1|CMC|HACO|IPCA|MCL|RNSC| 4 1.50021053443669 transport
YDL222C

YGL077C
YGR055W
YKL051W
>CHACO_C7098|2|CMC|HACO| 4 1.49782350460849 organelle organization
YAL029C
YGL106W
YHR023W
YOR326W
>CIPCA_C538|2|CL1|IPCA| 4 1.47264971149683 others
YIL002C
YKL212W
YNL106C
YOR109W
>CCMC_C203|2|CMC|HACO| 4 1.45781973107245 DNA metabolic process, response to stress
YHR164C
YKL113C
YMR190C
YOR033C
>CRNSC_C149|3|CMC|MCL|RNSC| 4 1.45258213020023 transport
YER136W
YFL005W
YFL038C
YOR370C
>CHACO_C7616|2|CMC|HACO| 4 1.42377019873833 transport, organelle organization
YGL180W
YLR423C
YPR049C
YPR185W
>CCMC_C257|3|CMC|IPCA|RNSC| 4 1.41063939864503 cell cycle process
YER179W
YHL022C
YIL072W
YPR007C
>CCMC_C80|2|CMC|RNSC| 11 1.37574733017993 protein metabolic process
YAL005C
YBL075C

YBR169C
YDL229W
YER103W
YLL024C
YMR186W
YNL007C
YNL064C
YNL209W
YPL106C
>CCMC_C124|2|CMC|IPCA| 10 1.36850204472387 RNA metabolic process
YBL026W
YCR077C
YDL160C
YEL015W
YER112W
YGL173C
YJL124C
YLR438C-A
YNL118C
YOL149W
>CCMC_C117|2|CMC|IPCA| 7 1.35943194044215 protein metabolic process
YBR170C
YBR201W
YDL126C
YGR048W
YLR207W
YML013W
YOL013C
>CCMC_C11|2|CMC|IPCA| 30 1.34407762100591 protein metabolic process
YBL041W
YBR173C
YDL007W
YDL097C
YDL147W
YDR394W
YER012W
YER021W
YER094C

YFL007W
YFR004W
YFR050C
YFR052W
YGL011C
YGL048C
YGR135W
YGR253C
YHL030W
YHR200W
YIL075C
YJL001W
YKL145W
YML092C
YMR314W
YOL038W
YOR157C
YOR261C
YOR362C
YPR103W
YPR108W
>CCMC_C167|2|CMC|IPCA| 6 1.32748570152108 organelle organization
YBR009C
YBR010W
YJL115W
YNL031C
YNL206C
YPR018W
>CCMC_C285|3|CMC|IPCA|RNSC| 5 1.31804729056999 transport
YBR068C
YCL025C
YEL063C
YKR039W
YPL265W
>CCL1_C179|2|CL1|IPCA| 4 1.30465740252201 response to chemical stimulus, regulation of gene expression, regulation of metabolic process
YER040W
YFL021W

YJL110C
YKR034W
>CHACO_C7675|2|HACO|RNSC| 4 1.29737104040876 transport
YBR267W
YGL099W
YHR170W
YIR026C
>CRNSC_C18|3|CL1|IPCA|RNSC| 4 1.29090761362805 organelle organization, response to stress
YLR332W
YNL283C
YOL105C
YOR008C
>CCL1_C160|3|CL1|IPCA|RNSC| 4 1.27776379177713 transport
YKL220C
YLR214W
YOL152W
YOR381W
>CHACO_C7672|2|CMC|HACO| 4 1.26409075459779 response to chemical stimulus, transport
YDL138W
YDL194W
YDR277C
YOR047C
>CCMC_C207|2|CMC|IPCA| 4 1.26022902360239 others
YDR309C
YHR061C
YLR229C
YNL298W
>CCMC_C148|2|CMC|IPCA| 6 1.2585459993084 others
YBL111C
YIL177C
YJL225C
YLL066C
YLL067C
YML133C
>CMCL_C207|2|CL1|MCL| 4 1.25640745975583 small molecule metabolic process

YBR196C
YGR087C
YLR044C
YLR134W
>CRNSC_C29|2|IPCA|RNSC| 5 1.25206796484103 others
YAL041W
YBR200W
YHL007C
YLR229C
YNL298W
>CMCL_C230|3|CL1|IPCA|MCL| 4 1.24440492067445 transport
YEL065W
YHL040C
YHL047C
YOL158C
>CCL1_C194|2|CL1|IPCA| 4 1.2344366352843 response to stress
YFL033C
YGL073W
YKL062W
YMR037C
>CRNSC_C105|2|MCL|RNSC| 4 1.22937362278277 regulation of gene expression, organelle organization, regulation of metabolic process
YDR227W
YKR101W
YLR442C
YNL216W
>CIPCA_C971|3|CL1|IPCA|MCL| 4 1.22669096788821 small molecule metabolic process
YCR034W
YDL015C
YJL196C
YLR372W
>CCMC_C138|2|CMC|IPCA| 12 1.22305882105864 RNA metabolic process
YDL043C
YDL209C
YER172C
YGL174W
YIR005W

YKL074C
YLL036C
YML049C
YMR213W
YMR240C
YMR288W
YPR101W
>CCL1_C215|2|CL1|IPCA| 4 1.22296529253952 small molecule metabolic process
YBR041W
YHR072W
YKL140W
YLL012W
>CCMC_C215|2|CMC|IPCA| 6 1.21389585263796 transport, organelle organization
YBL007C
YCR088W
YDR388W
YFR024C-A
YHR016C
YOR181W
>CCMC_C294|3|CMC|IPCA|RNSC| 4 1.19021804309307 response to chemical stimulus, response to stress
YDR353W
YGR209C
YLR043C
YPL091W
>CHACO_C9121|2|CMC|HACO| 4 1.18233310759941 DNA metabolic process, organelle organization
YDL220C
YDR082W
YLR233C
YLR318W
>CIPCA_C1160|3|CL1|IPCA|MCL|4 1.17068239181074 small molecule metabolic process
YCR053W
YDR158W
YER052C

YJR139C
>CIPCA_C419|3|CL1|IPCA|MCL| 5 1.17013061097571 protein metabolic process

YAL023C
YDL093W
YDL095W
YJR143C
YOR321W
>CCMC_C246|2|CMC|RNSC|5 1.16710622428913 organelle organization
YDL195W
YIL109C
YLR208W
YPL085W
YPR181C
>CIPCA_C1024|3|CL1|IPCA|MCL|4 1.12169104829698 DNA metabolic process, response to stress
YDR078C
YHL006C
YIL132C
YLR376C
>CCMC_C238|2|CMC|IPCA| 4 1.07060835880855 transport
YCL025C
YDL212W
YDR508C
YKR039W
>CMCL_C212|2|CL1|MCL| 4 1.06623294095123 transport
YDR038C
YDR039C
YDR040C
YLR138W
>CMCL_C189|2|IPCA|MCL| 4 1.05715296493887 others
YBR011C
YEL027W
YHR026W
YPL234C
>CCMC_C319|5|CL1|CMC|IPCA|MCL|RNSC| 15 1.05561130371854 others
YCR104W
YDR542W

YEL049W
YFL020C
YGL261C
YGR294W
YHL046C
YIL176C
YIR041W
YJL223C
YLL025W
YLL064C
YLR037C
YLR461W
YMR325W
>CMCL_C186|2|IPCA|MCL| 4 1.0256865939423 transport
YBR137W
YOL111C
YOR007C
YOR164C
>CCMC_C192|2|CMC|IPCA| 6 1.02300294683718 transport
YER110C
YJL041W
YLR293C
YLR335W
YLR347C
YMR308C
>CCMC_C311|3|CMC|IPCA|RNSC| 4 1.01330981879941 organelle organization
YBR168W
YDR479C
YHR150W
YLR324W
>CCMC_C183|2|CMC|IPCA| 6 1.00315796368219 organelle organization
YBL007C
YCR088W
YDR129C
YLR337C
YNL243W
YOR181W

>CHACO_C4330|1|HACO| 5 0.99924174758895 organelle organization
YAL011W
YBR231C
YDR485C
YLR085C
YML041C
>CHACO_C4596|1|HACO| 4 0.997833377035123 protein metabolic process, organelle organization
YBR111W-A
YGL066W
YMR223W
YPL047W
>CHACO_C5069|1|HACO| 5 0.99682685891519 protein metabolic process, organelle organization
YBL038W
YDR116C
YDR462W
YNL284C
YOR150W
>CHACO_C5151|1|HACO| 5 0.996082572963995 RNA metabolic process
YDL030W
YJL203W
YML049C
YMR240C
YOR319W
>CHACO_C5549|1|HACO| 8 0.994615218712863 protein metabolic process

YBR122C
YDR116C
YDR322W
YDR405W
YGR220C
YKL167C
YKR006C
YLR439W
>CMCL_C247|3|CL1|IPCA|MCL| 4 0.994329862137825 small molecule metabolic process
YML126C

YMR208W
YMR220W
YNR043W
>CHACO_C5956|1|HACO| 14 0.993632725761179 protein metabolic process

YBL027W
YBR181C
YBR191W
YDL075W
YDL082W
YDL136W
YDR471W
YGR034W
YHR141C
YJL177W
YKL180W
YMR194W
YNL096C
YNL301C
>CCMC_C278|2|CMC|IPCA| 4 0.992729061004031 DNA metabolic process, response to stress
YBR088C
YDL102W
YJR043C
YKL113C
>CMCL_C201|2|IPCA|MCL| 4 0.992606558814456 protein metabolic process

YBL071W-A
YIL103W
YKL191W
YLR172C
>CHACO_C5226|1|HACO| 4 0.992302054828772 transport, organelle organization
YAL030W
YMR183C
YOR327C
YPL232W
>CCMC_C274|2|CMC|IPCA| 5 0.990128891334926 others

YEL011W
YLR258W
YML100W
YMR105C
YPR160W
>CMCL_C209|2|CL1|MCL| 4 0.988950084115196 others
YAR071W
YBR092C
YBR093C
YHR215W
>CHACO_C6442|1|HACO| 12 0.98824039322985 RNA metabolic process
YDR087C
YER006W
YER126C
YGR103W
YHR052W
YKL009W
YKR081C
YMR049C
YNL002C
YOR272W
YPL093W
YPR016C
>CHACO_C5542|1|HACO| 4 0.988103579986946 RNA metabolic process
YKL172W
YLR196W
YOL077C
YOR206W
>CHACO_C6528|1|HACO| 7 0.986342859465057 protein metabolic process

YBR191W
YDL136W
YFR031C-A
YHR141C
YLR287C-A
YMR242C
YNL302C
>CHACO_C7054|1|HACO| 14 0.983488871848588 protein metabolic process

YBL072C
YBR048W
YDR025W
YDR447C
YER074W
YER102W
YHR203C
YIL069C
YJR145C
YLR441C
YML063W
YOR096W
YOR312C
YPL090C
>CHACO_C7594|1|HACO| 19 0.980411613776352 RNA metabolic process
YBL004W
YCR057C
YDL148C
YDR324C
YDR449C
YER082C
YGR128C
YHR148W
YHR196W
YJL109C
YJR002W
YLL011W
YLR186W
YLR409C
YMR093W
YPL126W
YPL217C
YPR137W
YPR144C
>CHACO_C7489|1|HACO| 38 0.979968023764 protein metabolic process
YBL027W
YBR031W

YBR048W
YBR181C
YBR191W
YCR031C
YDL075W
YDL082W
YDL083C
YDL136W
YDR012W
YDR418W
YDR471W
YER074W
YFR031C-A
YGL031C
YGL135W
YGL147C
YGR034W
YGR085C
YHL001W
YHL033C
YHR141C
YHR203C
YIL133C
YJL177W
YKL180W
YLL045C
YLR029C
YLR441C
YLR448W
YML063W
YML073C
YMR194W
YMR242C
YNL069C
YNL301C
YPL090C
>CHACO_C5832|1|HACO| 6 0.977761994842377 protein metabolic process, organelle organization

YDR036C
YDR041W
YDR347W
YIL093C
YNL137C
YPL118W
>CHACO_C6715|1|HACO| 10 0.977302048130676 RNA metabolic process
YDL030W
YDL043C
YER172C
YHR165C
YJL203W
YKL173W
YLL036C
YML049C
YMR240C
YPL213W
>CHACO_C6928|1|HACO| 11 0.976025721589159 protein metabolic process

YBR031W
YDR012W
YFR031C-A
YGL076C
YHL033C
YIL018W
YLL045C
YMR242C
YOL120C
YPL198W
YPL220W
>CHACO_C6628|1|HACO| 6 0.97515285086962 RNA metabolic process
YBR154C
YHR143W-A
YOR210W
YOR224C
YPR110C
YPR187W
>CHACO_C5869|1|HACO| 4 0.974848351376681 protein metabolic process

YGL076C
YMR142C
YOL120C
YPL198W
>CCL1_C46|2|CL1|IPCA| 9 0.974083556243223 organelle organization
YAL034W-A
YER018C
YGL093W
YIL144W
YIR010W
YJR112W
YMR117C
YOL069W
YPL233W
>CHACO_C6746|1|HACO| 13 0.970872491234062 protein metabolic process

YDR471W
YFR031C-A
YGL076C
YHL033C
YIL133C
YJL177W
YKL180W
YLL045C
YMR142C
YMR242C
YNL069C
YOR096W
YPL198W
>CHACO_C6760|1|HACO| 5 0.969726119742109 transport
YDL207W
YDR192C
YER107C
YKL068W
YMR047C
>CHACO_C6368|1|HACO| 6 0.967047762964891 RNA metabolic process
YDL209C

YDR482C
YLL036C
YMR213W
YPL151C
YPR101W
>CHACO_C6481|1|HACO| 4 0.964475612953575 RNA metabolic process
YBR142W
YDR060W
YOL041C
YPL043W
>CHACO_C7727|1|HACO| 21 0.962556809875852 protein metabolic process

YDL075W
YDL082W
YDR382W
YDR471W
YER074W
YGL030W
YGL103W
YIL133C
YJL177W
YKL180W
YLR075W
YLR441C
YLR448W
YML063W
YML073C
YMR194W
YNL069C
YNL096C
YOL127W
YOR063W
YOR096W
>CHACO_C7312|1|HACO| 9 0.962502927750998 RNA metabolic process
YDL148C
YDR449C
YER082C
YGR090W

YLR186W
YMR128W
YPL217C
YPR137W
YPR144C
>CRNSC_C163|2|CL1|RNSC| 7 0.961966846566939 DNA metabolic process
YBR098W
YBR228W
YDR386W
YLR135W
YLR234W
YMR190C
YPL024W
>CHACO_C6482|1|HACO| 8 0.960378959243689 protein metabolic process

YBL092W
YDR064W
YDR471W
YJL177W
YKL180W
YNL096C
YOL040C
YOR096W
>CHACO_C7315|1|HACO| 21 0.958901989095317 RNA metabolic process
YDL031W
YDR060W
YDR496C
YFL002C
YGL111W
YHR052W
YHR088W
YHR197W
YKR081C
YLL008W
YLL034C
YLR002C
YMR049C
YMR290C

YNL002C
YNL061W
YNL110C
YOL077C
YOR206W
YOR272W
YPL211W
>CHACO_C6882|1|HACO| 6 0.956061426099122 protein metabolic process

YBL084C
YDR113C
YGL003C
YGL116W
YHR166C
YKL022C
>CHACO_C7119|1|HACO| 13 0.954156191279912 RNA metabolic process
YAL032C
YDL209C
YDR416W
YER172C
YHR165C
YKL173W
YLL036C
YLR117C
YMR213W
YMR288W
YPL151C
YPL213W
YPR101W
>CHACO_C7103|1|HACO| 10 0.953479843586807 protein metabolic process

YFR004W
YFR010W
YHL030W
YHR027C
YHR200W
YIL075C
YKL145W

YLR421C
YOR117W
YOR261C
>CIPCA_C790|2|CL1|IPCA| 5 0.951656469322555 transport
YDR084C
YGL161C
YGL198W
YGR172C
YNL263C
>CMCL_C55|2|CL1|MCL| 9 0.947415742414654 transport
YDR069C
YDR486C
YKL002W
YKL041W
YKR035W-A
YLR025W
YLR181C
YPL084W
YPR173C
>CHACO_C6570|1|HACO| 4 0.946717671960936 protein metabolic process, organelle organization
YDR237W
YHR147C
YNL005C
YNL185C
>CHACO_C6981|1|HACO| 8 0.943412295098114 RNA metabolic process
YCL059C
YDL148C
YDR299W
YGR145W
YKR060W
YLR186W
YNL132W
YPR144C
>CHACO_C6899|1|HACO| 5 0.938214377923065 protein metabolic process

YBL075C
YBR169C

YDL229W
YNL209W
YPL106C
>CHACO_C6649|1|HACO| 5 0.935381398540717 RNA metabolic process
YBR247C
YDL060W
YKL143W
YNL207W
YOR056C
>CHACO_C7169|1|HACO| 10 0.932520889561453 RNA metabolic process
YAL032C
YDL209C
YDR364C
YDR416W
YGR278W
YKL095W
YLL036C
YMR213W
YPL151C
YPR101W
>CHACO_C7649|1|HACO| 17 0.932152720347453 RNA metabolic process
YCL054W
YDR087C
YER006W
YFR001W
YGR103W
YHR052W
YHR088W
YKL172W
YMR049C
YNL002C
YNL061W
YNL110C
YOL077C
YOR206W
YOR272W
YPL093W
YPL211W

>CMCL_C235|2|IPCA|MCL| 4 0.929149716809626 small molecule metabolic process
YDR400W
YLR017W
YLR209C
YNL129W
>CHACO_C6727|1|HACO| 6 0.925897267329599 transport
YDR159W
YER107C
YIL115C
YJL061W
YMR047C
YPL169C
>CMCL_C224|2|CL1|MCL| 4 0.920116998344724 organelle organization
YLL003W
YNL188W
YOL072W
YOR257W
>CHACO_C6987|1|HACO| 5 0.916878839783658 protein metabolic process

YGL030W
YGL103W
YHR010W
YOR063W
YPL249C-A
>CHACO_C7314|1|HACO| 8 0.901454039468347 RNA metabolic process
YDR449C
YER082C
YGR145W
YJL010C
YLL011W
YMR093W
YPL126W
YPL217C
>CHACO_C7656|1|HACO| 16 0.892291529215508 RNA metabolic process
YDR087C
YDR496C
YFR001W

YHR052W
YHR088W
YKL009W
YKL021C
YLR002C
YMR049C
YMR290C
YNL002C
YNL061W
YNL110C
YOR272W
YPL211W
YPR016C
>CHACO_C7164|1|HACO| 6 0.890701022833922 small molecule metabolic process
YGL012W
YGR060W
YGR175C
YHR007C
YLR056W
YMR015C
>CHACO_C7000|1|HACO| 4 0.888802129875525 others
YBL045C
YEL024W
YOR065W
YPR191W
>CHACO_C7392|1|HACO| 9 0.886688195033408 transport, organelle organization
YBL079W
YDL088C
YER105C
YFR002W
YLR018C
YML031W
YML103C
YMR129W
YMR153W
>CHACO_C6778|1|HACO| 4 0.88545924218809 protein metabolic process

YLR344W
YNL302C
YPL079W
YPR102C
>CHACO_C7632|1|HACO| 5 0.868144412107111 transport
YAR002W
YLR335W
YLR347C
YNL189W
YOR098C
>CHACO_C7358|1|HACO| 7 0.858400018043615 organelle organization
YDR190C
YGR002C
YJL081C
YLR385C
YLR399C
YNL107W
YPL235W
>CHACO_C7045|1|HACO| 5 0.852036736632144 regulation of metabolic process
YAL040C
YBR160W
YLR079W
YMR199W
YPL256C
>CRNSC_C110|4|CL1|IPCA|MCL|RNSC| 4 0.851568121055568 protein metabolic process
YFR041C
YLR090W
YNL077W
YPR061C
>CRNSC_C160|2|IPCA|RNSC| 6 0.850760918426004 others
YCL040W
YDR074W
YFR053C
YLR258W
YML100W
YMR105C

>CHACO_C7470|1|HACO| 5 0.849079400391778 protein metabolic process

YDL081C
YDL130W
YDR382W
YLR340W
YOL039W
>CMCL_C197|2|CL1|MCL| 4 0.84505897773917 others
YBL061C
YBR023C
YBR038W
YNL192W
>CCMC_C118|1|CMC| 4 0.844 transport
YDR381W
YGL122C
YKR095W
YPL169C
>CIPCA_C768|2|CL1|IPCA| 7 0.834974802414936 cell cycle process, DNA metabolic process, organelle organization
YCL016C
YHR191C
YMR048W
YMR078C
YNL273W
YPL008W
YPR135W
>CCMC_C122|1|CMC| 7 0.834 others
YDL065C
YDR244W
YDR329C
YGL153W
YLR191W
YOL044W
YOL147C
>CHACO_C7160|1|HACO| 7 0.832586116116853 organelle organization
YDL002C
YDR190C
YER092W

YFL013C
YGL150C
YLR052W
YPL235W
>CHACO_C7527|1|HACO| 8 0.830954701769088 protein metabolic process

YDR064W
YGL123W
YHL015W
YJR123W
YNL178W
YOL040C
YOR369C
YPL131W
>CHACO_C7210|1|HACO| 5 0.830654059240482 transport, organelle organization
YBR091C
YEL020W-A
YGR181W
YHR005C-A
YJR135W-A
>CCMC_C305|2|CMC|IPCA| 4 0.825702619226577 organelle organization
YDR379W
YLR229C
YOR127W
YPL115C
>CCMC_C41|1|CMC| 5 0.824 DNA metabolic process, cell cycle process, organelle organization
YDL003W
YFL008W
YFR031C
YJL074C
YLR086W
>CCMC_C131|1|CMC| 4 0.823 regulation of gene expression, regulation of metabolic process, RNA metabolic process
YBR245C
YER164W
YOR304W

YPL082C
>CIPCA_C1306|1|IPCA| 30 0.82259423511287 protein metabolic process
YBL027W
YBL092W
YBR181C
YBR191W
YDL075W
YDL082W
YDL136W
YDR064W
YDR447C
YDR471W
YER074W
YFR031C-A
YFR032C-A
YGL014W
YGL103W
YGR034W
YHL001W
YHR141C
YJL177W
YLL013C
YLR287C-A
YLR448W
YML063W
YML073C
YMR194W
YNL096C
YNL301C
YNL302C
YOL040C
YOL127W
>CCMC_C139|1|CMC| 8 0.817 RNA metabolic process
YCR072C
YGR245C
YHR085W
YHR197W
YLR106C

YNL182C
YNR053C
YPL093W
>CRNSC_C76|1|RNSC| 11 0.81537960845545 RNA metabolic process
YBR119W
YDL087C
YDR235W
YDR240C
YGR013W
YHR086W
YIL061C
YKL012W
YLR298C
YPL178W
YPR057W
>CCMC_C141|1|CMC| 4 0.81 transport, organelle organization
YBL007C
YNL084C
YNL243W
YOR181W
>CCMC_C144|1|CMC| 4 0.809 protein metabolic process, response to stress

YCR073C
YJL128C
YLR006C
YNR031C
>CCMC_C291|2|CMC|IPCA| 4 0.80791796652265 DNA metabolic process, cell cycle process, response to stress
YML032C
YML095C
YOL090W
YPL022W
>CIPCA_C1191|2|IPCA|MCL| 4 0.800785191530169 small molecule metabolic process
YGR286C
YNR056C
YNR057C
YNR058W

>CCMC_C301|2|CMC|IPCA| 4 0.798587237257367 transport
YDL192W
YER122C
YIL004C
YLR268W
>CMCL_C137|1|MCL| 5 0.796482556502115 organelle organization
YBR010W
YDR225W
YNL030W
YNL031C
YNL206C
>CCL1_C60|2|CL1|IPCA| 9 0.786196682425898 transport
YBR171W
YBR283C
YDR086C
YER019C-A
YER087C-B
YLR292C
YLR378C
YOR254C
YPL094C
>CIPCA_C878|2|CL1|IPCA| 4 0.783100321280741 response to stress
YDR294C
YJL134W
YLR260W
YOR171C
>CCMC_C320|4|CMC|IPCA|MCL|RNSC| 4 0.778851811101784 others
YEL075C
YER189W
YFL064C
YLR462W
>CIPCA_C503|2|IPCA|RNSC| 4 0.777634623556342 protein metabolic process, response to chemical stimulus, response to stress
YJL034W
YJL073W
YKL073W
YMR214W
>CMCL_C214|2|IPCA|MCL| 4 0.777001241637702 RNA metabolic process

YDR267C
YGL091C
YIL003W
YNL240C
>CCMC_C169|1|CMC| 5 0.777 protein metabolic process, organelle organization
YDL045W-A
YDR175C
YIL093C
YJR113C
YNL081C
>CCMC_C307|2|CMC|RNSC|4 0.776467766124942 RNA metabolic process
YDL063C
YIL096C
YLR051C
YOL022C
>CHACO_C7604|1|HACO| 9 0.775999945574814 others
YCR072C
YDR101C
YGR245C
YJL122W
YKL009W
YLR009W
YLR074C
YNR053C
YPR016C
>CHACO_C7264|1|HACO| 11 0.773155734368794 protein metabolic process

YDL061C
YDR450W
YGL031C
YGR118W
YKR094C
YLR388W
YNL067W
YNL302C
YOR234C
YPL143W

YPR102C
>CCL1_C42|2|CL1|IPCA| 5 0.7597591215453 others
YIL011W
YJR151C
YNR076W
YOR009W
YOR010C
>CCMC_C196|1|CMC| 4 0.745 DNA metabolic process, response to stress
YBR136W
YBR274W
YDR217C
YPL153C
>CCMC_C200|1|CMC| 5 0.734 protein metabolic process
YAL005C
YDR172W
YLL026W
YNL007C
YNL064C
>CCMC_C112|1|CMC| 5 0.727 cell cycle process, organelle organization
YBR156C
YGL061C
YGR113W
YKR037C
YPL209C
>CRNSC_C4|2|IPCA|RNSC| 4 0.725881974705104 regulation of gene expression, small molecule metabolic process, regulation of metabolic process
YGR180C
YJL026W
YOR229W
YOR230W
>CCMC_C206|1|CMC| 4 0.725 protein metabolic process, regulation of gene expression, organelle organization, regulation of metabolic process, RNA metabolic process
YCR060W
YDR190C
YHR034C
YPL235W
>CHACO_C9091|1|HACO| 6 0.71851013159379 protein metabolic process

YBR189W
YDR450W
YIL069C
YJL190C
YOR204W
YPL081W
>CHACO_C7731|1|HACO| 5 0.71831146836153 DNA metabolic process
YDR004W
YDR076W
YER095W
YGL163C
YML032C
>CHACO_C7578|1|HACO| 7 0.716210919623961 protein metabolic process

YDL061C
YDR450W
YDR500C
YGR214W
YLR048W
YLR388W
YPL143W
>CCL1_C233|2|CL1|IPCA| 5 0.716161266949434 others
YBR024W
YBR037C
YER141W
YPL132W
YPL172C
>CRNSC_C138|1|RNSC| 4 0.714774323326384 RNA metabolic process
YCR063W
YNL138W-A
YOR319W
YPR094W
>CIPCA_C899|2|CL1|IPCA| 4 0.713733724340658 small molecule metabolic process
YER073W
YMR169C
YMR170C
YOR374W

>CIPCA_C330|2|IPCA|RNSC|6 0.70943640765383 small molecule metabolic process
YCL009C
YJR016C
YJR148W
YLR355C
YMR108W
YNL104C
>CCMC_C193|1|CMC| 11 0.709 RNA metabolic process
YBR142W
YDL031W
YFL002C
YGL078C
YGL171W
YHR065C
YHR169W
YKR024C
YLL008W
YLR276C
YMR290C
>CCMC_C219|1|CMC| 7 0.708 protein metabolic process
YBR173C
YGR135W
YGR253C
YLR021W
YOR157C
YPL144W
YPR103W
>CCMC_C96|1|CMC| 5 0.705 DNA metabolic process, response to stress
YBR088C
YCR066W
YDR092W
YJL092W
YLR032W
>CCMC_C225|1|CMC| 4 0.701 transport
YDR142C
YGR239C
YHR160C

YLR191W
>CCMC_C105|1|CMC| 12 0.7 organelle organization
YBR245C
YCR052W
YDR303C
YFR037C
YIL126W
YKR001C
YLR033W
YLR357W
YMR033W
YMR091C
YPL082C
YPR052C
>CHACO_C9115|1|HACO| 8 0.69773777280053 protein metabolic process
YDL061C
YER131W
YGR027C
YLR333C
YLR388W
YNL067W
YNL302C
YOR293W
>CCMC_C226|1|CMC| 6 0.696 RNA metabolic process
YBR142W
YKL014C
YLL008W
YLR276C
YMR290C
YNR038W
>CCMC_C306|2|CMC|IPCA| 5 0.694058819940068 others
YBR271W
YGR283C
YIL096C
YMR310C
YNL022C
>CRNSC_C60|2|IPCA|RNSC| 6 0.689581887289274 RNA metabolic process
YBR061C

YDL201W
YDR120C
YDR165W
YKR056W
YOL124C
>CIPCA_C792|2|CL1|IPCA| 6 0.68904639265139 cell cycle process
YBL063W
YEL061C
YGL216W
YKR054C
YPL155C
YPR141C
>CCMC_C62|1|CMC| 6 0.689 transport
YDL116W
YDR159W
YDR381W
YJR042W
YKL186C
YPL169C
>CIPCA_C1307|2|IPCA|MCL| 4 0.686140540677774 protein metabolic process
YDL230W
YER075C
YNL053W
YOR208W
>CCMC_C121|1|CMC| 6 0.684 regulation of metabolic process
YAL040C
YBR160W
YDL056W
YER111C
YLR182W
YPL256C
>CRNSC_C150|1|RNSC| 15 0.683288509112428 RNA metabolic process
YDL115C
YDL140C
YDR404C
YER139C
YGL043W

YGL070C
YGR005C
YGR063C
YGR186W
YHR143W-A
YIL021W
YJL140W
YML010W
YOL005C
YOR151C
>CIPCA_C1354|1|IPCA| 43 0.680546634964468 protein metabolic process
YBL027W
YBL072C
YBL087C
YBR031W
YBR181C
YDL075W
YDL082W
YDL083C
YDL191W
YDR012W
YDR064W
YDR418W
YDR471W
YER074W
YGL030W
YGL076C
YGL103W
YGL135W
YGR034W
YHL001W
YHL033C
YHR010W
YJL177W
YLL045C
YLR075W
YLR406C
YLR441C

YML073C
YMR142C
YMR242C
YMR290C
YNL069C
YNL110C
YNL301C
YNL313C
YOL127W
YOR063W
YOR312C
YPL090C
YPL131W
YPL143W
YPL249C-A
YPR102C
>CCMC_C234|1|CMC| 5 0.678 others
YAL055W
YDR265W
YGR133W
YNL329C
YOL044W
