# Supplementary material for: Supervised maximum-likelihood weighting of composite protein networks for complex prediction
Source: BMC Syst Biol. 2012 Dec 12;6(Suppl 2):S13. doi: 10.1186/1752-0509-6-S2-S13 (PMC3521185; doi:10.1186/1752-0509-6-S2-S13)
Supplement: Additional file 2 — Novel, unique, high-confidence predicted human complexes. [file 1752-0509-6-S2-S13-S2.pdf]

>CCMC_C0|6|CL1|CMC|HACO|IPCA|MCL|RNSC| 4 5.4515690155182 transport
O60741
Q9P1Z3
Q9UL51
Q9Y3Q4
>CHACO_C8585|5|CL1|CMC|HACO|IPCA|MCL| 4 4.14197976042419 regulation of gene expression, regulation of metabolic process, RNA metabolic process
Q9H9G7
Q9HCK5
Q9UKV8
Q9UL18
>CHACO_C10994|6|CL1|CMC|HACO|IPCA|MCL|RNSC| 7 3.9177901371611 organelle organization
O94927
Q7Z4H7
Q96CS2
Q99871
Q9BT25
Q9H6D7
Q9NVX0
>CCMC_C116|6|CL1|CMC|HACO|IPCA|MCL|RNSC| 4 3.72262558716671 regulation of gene expression, regulation of metabolic process
O00287
O14593
P33076
P48382
>CCMC_C11|5|CL1|CMC|HACO|IPCA|RNSC| 4 3.57519793242791 response to stress
P13498
P14598
P19878
Q15080
>CHACO_C14096|6|CL1|CMC|HACO|IPCA|MCL|RNSC| 9 3.43072619407341 regulation of metabolic process
O00238
P12643
P12644

P18075
P22004
P36894
P43026
Q13873
Q6KF10
>CHACO_C8508|5|CL1|CMC|HACO|IPCA|RNSC| 4 3.42049183816081
regulation of gene expression, regulation of metabolic process
P28360
P35548
P56178
Q07687
>CCMC_C181|6|CL1|CMC|HACO|IPCA|MCL|RNSC| 4 3.31106425101667
regulation of metabolic process
O75293
O95257
P24522
Q9Y6R4
>CCL1_C400|6|CL1|CMC|HACO|IPCA|MCL|RNSC| 4 3.13044254175803 others

O00339
O15232
O95460
P21941
>CCMC_C125|5|CMC|HACO|IPCA|MCL|RNSC| 4 3.08367555660549 organelle organization
P56282
Q07864
Q9NR33
Q9NRF9
>CHACO_C10504|6|CL1|CMC|HACO|IPCA|MCL|RNSC| 4 3.01636815776957
others
O43399
P55327
Q16890
Q96J77
>CHACO_C10780|5|CL1|CMC|HACO|IPCA|RNSC| 8 2.93256141519568 cell cycle process, regulation of metabolic process

O60216
Q29RF7
Q7Z5K2
Q8N3U4
Q8TCV5
Q8WVM7
Q9NTI5
Q9UQE7
>CCMC_C50|4|CMC|HACO|MCL|RNSC| 6 2.83329570839222 others
P32926
Q02413
Q02487
Q08554
Q14126
Q14574
>CHACO_C11980|6|CL1|CMC|HACO|IPCA|MCL|RNSC| 6 2.76858501005079 RNA metabolic process
P17480
Q15572
Q15573
Q53T94
Q9H5J8
Q9NYV6
>CRNSC_C343|5|CL1|CMC|HACO|IPCA|RNSC| 4 2.71469553860065 regulation of gene expression, regulation of metabolic process
P08151
P10070
P10071
Q9UMX1
>CHACO_C11954|5|CL1|CMC|HACO|IPCA|RNSC| 6 2.69486203893577 cell cycle process, organelle organization
P23258
Q08AG7
Q6NZ67
Q96CW5
Q96RT8
Q9BSJ2
>CCL1_C610|4|CL1|CMC|HACO|IPCA|5 2.68284875346503 regulation of

metabolic process
P00533
P01133
P04626
P21860
Q15303
>CCL1_C153|6|CL1|CMC|HACO|IPCA|MCL|RNSC| 6 2.59209843862848 DNA metabolic process, regulation of metabolic process, response to stress
P46736
Q15018
Q6UWZ7
Q96RL1
Q9NWV8
Q9NXR7
>CHACO_C14698|6|CL1|CMC|HACO|IPCA|MCL|RNSC| 7 2.55410711919938 response to chemical stimulus
P02795
P04732
P04733
P80294
P80297
Q8N339
Q93083
>CCMC_C2|3|CMC|HACO|RNSC| 4 2.50204734289223 transport
P16389
P22001
P22459
Q09470
>CIPCA_C850|6|CL1|CMC|HACO|IPCA|MCL|RNSC| 4 2.47893368261563 others

A5PLN9
O43617
Q9UL33
Q9Y2L5
>CCMC_C56|4|CL1|CMC|HACO|RNSC| 4 2.47361345674462 regulation of gene expression, regulation of metabolic process
P45973
P83916

Q13185
Q13263
>CHACO_C9099|4|CMC|HACO|IPCA|RNSC| 4 2.45518009601125 response to chemical stimulus
P02461
P05997
P08123
P20908
>CCMC_C178|4|CMC|HACO|MCL|RNSC| 4 2.40753492810519 response to stress
Q14164
Q14653
Q92844
Q9UHD2
>CCL1_C103|4|CL1|IPCA|MCL|RNSC| 11 2.32274313831483 cell cycle process
O14777
O43683
O95229
Q6P1K2
Q8NBT2
Q8NG31
Q96IY1
Q9BZD4
Q9H081
Q9H410
Q9HBM1
>CHACO_C15207|5|CL1|CMC|HACO|IPCA|RNSC| 4 2.31680031736143 transport, small molecule metabolic process
O00194
P51159
Q9BV36
Q9Y2J0
>CHACO_C10421|5|CL1|CMC|HACO|IPCA|RNSC| 5 2.31543519262623 transport
O15155
O15498
O60763
O95249

Q13190
>CHACO_C11017|5|CL1|CMC|HACO|IPCA|RNSC| 4 2.31503855557191 cell cycle process
O15078
O43303
Q5T7B8
Q8TAP6
>CHACO_C12127|5|CL1|CMC|HACO|IPCA|RNSC| 4 2.28875675095888 others

P78411
P78414
P78415
Q9BZI1
>CCMC_C420|5|CL1|CMC|HACO|IPCA|RNSC| 4 2.25921217362421 protein metabolic process
P05198
P20042
P41091
P43146
>CRNSC_C86|5|CL1|CMC|HACO|IPCA|RNSC| 5 2.23580034643659 response to chemical stimulus
P01903
P02686
P13760
P28067
P28068
>CRNSC_C143|5|CL1|CMC|HACO|IPCA|RNSC| 4 2.18597746388923 regulation of gene expression, regulation of metabolic process
P10747
P16410
P33681
P42081
>CRNSC_C17|4|CL1|IPCA|MCL|RNSC| 9 2.1818148302797 response to chemical stimulus
P20591
P48995
Q13507
Q14573

Q14643
Q9HCX4
Q9UBN4
Q9UL62
Q9Y210
>CCL1_C158|3|CL1|CMC|HACO| 4 2.17256977941125 regulation of metabolic process
P27037
P36896
Q04771
Q13705
>CCMC_C454|5|CL1|CMC|HACO|IPCA|RNSC| 6 2.14991328964058 response to chemical stimulus
O14531
Q16827
Q7L190
Q99961
Q99962
Q99963
>CRNSC_C82|5|CL1|CMC|HACO|IPCA|RNSC| 4 2.04689089489755 others
P09067
P14651
P14652
P17509
>CHACO_C9919|4|CMC|HACO|IPCA|RNSC| 4 2.04308514689576 regulation of gene expression, regulation of metabolic process
P20719
P31260
P31268
P31269
>CIPCA_C10|4|CL1|IPCA|MCL|RNSC| 21 1.96758675880994 protein metabolic process
O43684
O60566
O95996
O95997
P12830
P14635

P30260
Q12834
Q13042
Q8NHZ8
Q96DE5
Q9BS18
Q9H1A4
Q9NYG5
Q9UJX2
Q9UJX3
Q9UJX4
Q9UJX5
Q9UJX6
Q9UM11
Q9UM13
>CHACO_C13811|5|CL1|CMC|HACO|IPCA|RNSC| 6 1.96149102491669 response to chemical stimulus
Q5VZM2
Q7L523
Q9HB90
Q9NQL2
Q9UHA4
Q9Y2Q5
>CCMC_C339|4|CMC|HACO|IPCA|RNSC| 4 1.92817030379985 transport
O43633
Q96FZ7
Q9BY43
Q9Y3E7
>CHACO_C10420|4|CL1|CMC|HACO|RNSC| 5 1.92358321991031 organelle organization
O15260
P49755
Q15363
Q9BVK6
Q9Y3B3
>CCMC_C428|5|CL1|CMC|HACO|IPCA|RNSC| 4 1.91449887289875 others
O43707
O60500

Q9NP85
Q9Y5K6
>CHACO_C11898|5|CL1|CMC|HACO|IPCA|RNSC| 4 1.78314534388858 transport
P15382
P51787
Q12809
Q9Y6J6
>CRNSC_C3|4|CL1|CMC|IPCA|RNSC| 4 1.77322073262136 others
O60282
P33176
Q07866
Q9H0B6
>CCMC_C360|4|CMC|HACO|IPCA|RNSC| 4 1.76255561483895 transport
O15438
O15439
O15440
Q92887
>CMCL_C293|6|CL1|CMC|HACO|IPCA|MCL|RNSC| 5 1.76049198195972 response to chemical stimulus
P30872
P30874
P31391
P32745
P35346
>CCMC_C369|4|CL1|CMC|HACO|MCL| 4 1.75910829579763 protein metabolic process, transport, organelle organization
O94855
O95487
P53992
Q15436
>CCL1_C160|3|CL1|IPCA|RNSC| 6 1.74852409856064 RNA metabolic process
A5YKK6
O75175
Q96LI5
Q9NZN8
Q9UFF9
Q9ULM6

>CCMC_C378|4|CL1|CMC|IPCA|RNSC| 7 1.72196115649121 others
P50749
Q13043
Q13188
Q8WWW0
Q9H2L5
Q9H4B6
Q9NS23
>CHACO_C14835|5|CL1|CMC|HACO|IPCA|RNSC| 6 1.66227946571467 others

O00743
O15084
Q5H9R7
Q70CQ1
Q8NB46
Q9UPN7
>CRNSC_C90|5|CL1|CMC|HACO|IPCA|RNSC| 4 1.6292364695832 others
Q6P9H5
Q8WWP7
Q96F15
Q9NUV9
>CHACO_C11998|3|CMC|HACO|IPCA|4 1.61917590826696 regulation of gene expression, regulation of metabolic process, response to stress
P01100
P05412
P15336
P18847
>CIPCA_C435|4|CL1|IPCA|MCL|RNSC| 6 1.61300051048149 regulation of gene expression, regulation of metabolic process
Q13422
Q9BZS1
Q9H2S9
Q9H5V7
Q9UKS7
Q9UKT9
>CCMC_C193|3|CMC|HACO|RNSC| 7 1.6124296474071 protein metabolic process, regulation of metabolic process
P46060

P55854
P61956
P63165
P63279
Q9UBE0
Q9UBT2
>CCMC_C548|5|CL1|CMC|HACO|IPCA|RNSC| 4 1.60835568469854 transport, regulation of gene expression, regulation of metabolic process
P06748
P19338
P32121
P49407
>CCMC_C422|4|CMC|HACO|IPCA|RNSC| 4 1.60162656476597 transport
O14745
P13569
Q15599
Q5T2W1
>CCMC_C293|3|CMC|IPCA|RNSC| 4 1.58332835045425 transport
P37088
P46934
P51168
P51170
>CRNSC_C268|4|CL1|IPCA|MCL|RNSC| 7 1.5784042370694 others
O60346
O75317
P62068
Q09019
Q6ZVD8
Q8TAF3
Q8TBZ3
>CHACO_C9968|3|CMC|HACO|RNSC| 4 1.57607437638686 small molecule metabolic process
P09210
P21266
P28161
Q03013
>CHACO_C13821|3|CMC|HACO|MCL| 4 1.57147069814079 regulation of gene expression, regulation of metabolic process

P41134
Q02363
Q02535
Q96BH3
>CRNSC_C211|4|CL1|IPCA|MCL|RNSC| 4 1.56291095106797 others
Q13586
Q96D31
Q96SN7
Q9P246
>CCMC_C303|3|CMC|HACO|RNSC| 4 1.5627507325215 RNA metabolic process
O14802
P05423
Q9NVU0
Q9Y535
>CHACO_C8184|2|CMC|HACO| 5 1.5514668863673 RNA metabolic process
O75533
Q13435
Q15393
Q15427
Q15428
>CRNSC_C217|4|CL1|IPCA|MCL|RNSC| 7 1.53081667138327 others
O60941
P11532
P46939
Q13424
Q13425
Q13884
Q9Y4J8
>CCMC_C580|5|CL1|CMC|IPCA|MCL|RNSC| 4 1.52934837318826 regulation of gene expression, regulation of metabolic process
P20823
P61457
Q9H0N5
Q9Y463
>CRNSC_C318|3|CL1|IPCA|RNSC| 19 1.52094991363315 cell cycle process
O00311
O43913

O43929
O75419
P15927
P25205
P33991
P33992
P33993
P49736
Q13415
Q13416
Q14566
Q7L590
Q99741
Q9H211
Q9UBD5
Q9UBU7
Q9Y5N6
>CIPCA_C226|4|CL1|IPCA|MCL|RNSC| 7 1.47408104407444 response to stress
Q14457
Q6ZNE5
Q8NEB9
Q92622
Q96F24
Q99570
Q9P2Y5
>CHACO_C8958|2|CMC|HACO| 4 1.46689118093103protein metabolic process, organelle organization
O94817
O95352
Q9H1Y0
Q9NT62
>CCMC_C6|2|CMC|IPCA| 8 1.45314679115488 protein metabolic process, cell cycle process, small molecule metabolic process, RNA metabolic process, regulation of metabolic process, response to stress
P20618
P25787
P28072

P28074
P40306
P49720
P49721
Q99436
>CCMC_C526|4|CMC|HACO|MCL|RNSC| 4 1.43345373626589 transport
O15533
P30443
Q03518
Q03519
>CHACO_C13331|4|CMC|HACO|IPCA|RNSC| 4 1.41485421269459 regulation of metabolic process
O00222
P41594
Q14416
Q14833
>CIPCA_C2845|3|CL1|IPCA|MCL|4 1.41405713939927 regulation of gene expression, regulation of metabolic process
O15119
P43694
P52952
Q99593
>CCMC_C21|2|CMC|IPCA| 9 1.39528289454288 cell cycle process
O00311
O75419
P33991
P33993
P49736
Q13415
Q13416
Q9H211
Q9UBU7
>CRNSC_C156|4|CL1|IPCA|MCL|RNSC| 6 1.38590033533951 regulation of gene expression, regulation of metabolic process
O15055
O15534
P56645
Q16526

Q49AN0
Q99743
>CHACO_C9784|2|CMC|HACO| 4 1.3826675762827 transport
Q05586
Q12879
Q13224
Q14957
>CHACO_C12555|2|CMC|HACO| 4 1.38025236620191 transport, RNA metabolic process
Q01130
Q05519
Q07955
Q13243
>CCMC_C313|3|CMC|IPCA|RNSC| 7 1.37977319834834 transport
O00560
O43424
P42261
P42262
Q13002
Q16478
Q9NRD5
>CHACO_C11321|2|CMC|HACO| 6 1.37833817849643 transport
Q3SYG4
Q8IWZ6
Q8N3I7
Q8NFJ9
Q96RK4
Q9BXC9
>CHACO_C10605|2|CMC|HACO| 6 1.37671610079994 organelle organization, response to chemical stimulus
O43521
P10415
Q07817
Q07820
Q16611
Q92934
>CCL1_C403|3|CL1|IPCA|MCL| 4 1.37372765545708protein metabolic process, response to chemical stimulus, regulation of gene expression, regulation of

metabolic process, response to stress
P49770
Q13144
Q14232
Q9NR50
>CRNSC_C294|4|CMC|IPCA|MCL|RNSC| 4 1.37221114195169 response to chemical stimulus
P12107
P13942
P20849
Q14055
>CRNSC_C50|4|CL1|IPCA|MCL|RNSC| 4 1.34250430695702 small molecule metabolic process
O60488
O95573
P33121
Q9ULC5
>CCMC_C244|3|CL1|CMC|IPCA| 4 1.33393727351888 regulation of metabolic process
P00734
P00749
P00750
P05121
>CRNSC_C266|4|CL1|IPCA|MCL|RNSC| 4 1.3331914862444 regulation of gene expression, regulation of metabolic process
Q86SG3
Q92904
Q96EP5
Q9NQZ3
>CCMC_C338|3|CMC|IPCA|RNSC| 7 1.32365199162106 response to chemical stimulus
O60716
P12830
P14923
P19022
P33151
P35221
P35222

>CRNSC_C178|5|CMC|HACO|IPCA|MCL|RNSC| 4 1.32124926692397 RNA metabolic process
O60832
Q9NPE3
Q9NX24
Q9NY12
>CCMC_C440|3|CMC|IPCA|RNSC| 4 1.31633215393642 small molecule metabolic process
P00395
P00403
P00414
P03928
>CRNSC_C305|4|CL1|IPCA|MCL|RNSC| 6 1.31274641099674 protein metabolic process, regulation of metabolic process
Q8IXJ6
Q8N6T7
Q9NRC8
Q9NTG7
Q9NXA8
Q9Y6E7
>CHACO_C9018|2|HACO|MCL| 4 1.30425473887437protein metabolic process, regulation of metabolic process
P42574
P55210
P55211
P55212
>CRNSC_C73|3|CL1|IPCA|RNSC| 4 1.29399261058605protein metabolic process, DNA metabolic process, cell cycle process, regulation of metabolic process
O14757
P30304
P30305
P30307
>CMCL_C441|4|CL1|IPCA|MCL|RNSC|4 1.29383392340174 response to chemical stimulus, regulation of metabolic process, response to stress
O00585
O43927
P32248
P32302

>CHACO_C12016|2|CMC|HACO| 7 1.29301920262596protein metabolic process, transport, RNA metabolic process
P18124
P30050
P36578
P39023
P46778
P83731
Q02878
>CHACO_C12018|2|CMC|HACO| 4 1.2925651972778 organelle organization
P02533
P08779
P13645
P13647
>CHACO_C15323|5|CL1|CMC|HACO|IPCA|RNSC| 6 1.29178901941955 others

O60861
Q7L576
Q92558
Q96F07
Q9Y2A7
Q9Y6W5
>CCMC_C299|3|CMC|IPCA|RNSC| 6 1.29055647886294 regulation of metabolic process
O43741
P54619
P54646
Q8IYT8
Q9UGJ0
Q9Y478
>CRNSC_C177|3|CL1|IPCA|RNSC| 7 1.28834600881596 others
P57735
P62491
Q15907
Q6WKZ4
Q7L804
Q86YS3
Q9BXF6

>CMCL_C119|4|CL1|IPCA|MCL|RNSC|7 1.28081226366048 others
P06731
P13688
P31997
P40198
P40199
Q14002
Q16568
>CHACO_C11394|3|CMC|HACO|RNSC| 4 1.27674344475232 regulation of gene expression, regulation of metabolic process
P13378
P28356
P28358
P31277
>CRNSC_C161|3|IPCA|MCL|RNSC| 4 1.26837046164908 cell cycle process, response to chemical stimulus, regulation of metabolic process
Q15831
Q7RTN6
Q9C0K7
Q9Y376
>CCMC_C400|3|CMC|IPCA|RNSC| 4 1.26644706629658 transport
P14416
P21917
P48544
P48549
>CCMC_C119|2|CMC|RNSC|7 1.26036427252795 regulation of metabolic process
P11802
P24385
P24941
P30279
P38936
P46527
Q00534
>CCMC_C473|3|CMC|HACO|MCL| 4 1.25672782763684 protein metabolic process, transport
O76094
P37108

P49458
Q9UHB9
>CCMC_C647|5|CL1|CMC|IPCA|MCL|RNSC| 4 1.24248118321222 others
Q8IZL8
Q9BV38
Q9H4L4
Q9NXF1
>CCMC_C435|3|CMC|IPCA|RNSC| 11 1.24094509025381 others
O14763
P15311
P25445
P26038
P45983
P48023
P52565
P55957
P61586
Q13158
Q92851
>CHACO_C10383|2|CMC|HACO| 9 1.23343841483731 others
O43815
P30153
P30154
P62714
P67775
Q13033
Q9BRV8
Q9NRL3
Q9Y3A3
>CIPCA_C598|4|CL1|IPCA|MCL|RNSC| 5 1.22747446092348 others
P10321
P43626
P43628
Q14954
Q8N743
>CRNSC_C290|4|CL1|IPCA|MCL|RNSC| 4 1.21805731871933 transport
P21754
P60852

Q05996
Q12836
>CRNSC_C152|4|CL1|IPCA|MCL|RNSC| 10 1.20533988877014 transport
O14520
O43315
O94778
P29972
P41181
P55064
P55087
Q13520
Q92482
Q96PS8
>CHACO_C12617|2|CMC|HACO| 4 1.20383511510732protein metabolic process, response to stress
P01834
P01857
P01876
P02787
>CHACO_C11509|2|CMC|HACO| 4 1.20216604160987 transport, small molecule metabolic process
P06576
P24539
P25705
P36542
>CCMC_C183|2|CMC|RNSC|4 1.19760514437823 transport, response to chemical stimulus
P00734
P02671
P02675
P02679
>CMCL_C70|3|CL1|MCL|RNSC| 9 1.18624514706695 transport
O00623
O00628
O60683
O75381
P40855
P50542

P56589
Q92968
Q9Y5Y5
>CIPCA_C844|2|CMC|IPCA| 4 1.17445250378571 others
P51114
P51116
Q06787
Q7L576
>CCMC_C91|2|CMC|IPCA| 5 1.17247944849969 others
P08962
P19397
P21926
P27701
P48509
>CIPCA_C1007|3|CL1|IPCA|MCL|4 1.16964210860385 protein metabolic process

Q14118
Q8WZA1
Q9UKY4
Q9Y6A1
>CCMC_C123|2|CMC|IPCA| 4 1.16682308465464 others
P02489
P02511
P07315
P43320
>CHACO_C12936|2|CMC|HACO| 4 1.15661432143797 protein metabolic process

P06241
P08631
P12931
P41240
>CCL1_C30|3|CL1|IPCA|RNSC| 18 1.15071099781319 protein metabolic process

P17987
P40227
P48643
P49368
P50990

P50991
P60510
P62714
P63151
P67775
P78318
P78371
Q00005
Q15257
Q66LE6
Q99832
Q9H7D0
Q9Y2T4
>CCMC_C811|6|CL1|CMC|HACO|IPCA|MCL|RNSC| 4 1.15052907313123 regulation of gene expression, regulation of metabolic process, RNA metabolic process
O00712
P08651
P22670
Q14938
>CHACO_C9637|2|CMC|HACO| 7 1.14546403904572 others
O00506
Q9BRV8
Q9BUL8
Q9NRL3
Q9P289
Q9Y228
Q9Y6E0
>CHACO_C11523|2|CMC|HACO| 5 1.14220874933981 others
P41743
P60953
Q9BYG4
Q9BYG5
Q9NPB6
>CCMC_C469|3|CMC|HACO|IPCA| 4 1.13678380049494 small molecule metabolic process
P08684
P10632

P11712
P20813
>CCMC_C234|2|CMC|HACO| 5 1.13171463940362 organelle organization
O00401
O43516
Q9BY11
Q9UKS6
Q9UNF0
>CHACO_C11975|2|CMC|HACO| 4 1.12171245077781 response to chemical stimulus, regulation of metabolic process, response to stress
P19438
Q13546
Q15628
Q9Y572
>CMCL_C405|4|CL1|IPCA|MCL|RNSC|4 1.11599161465458 protein metabolic process
O94874
P61960
Q9GZZ9
Q9Y3C8
>CCMC_C118|2|CMC|IPCA| 4 1.11377341130575 protein metabolic process, regulation of gene expression, regulation of metabolic process, response to stress
P27361
P28482
P45983
Q16539
>CHACO_C14401|3|CMC|HACO|RNSC| 4 1.11352592628725 others
O43240
P49862
Q9UBX7
Q9Y337
>CRNSC_C40|3|CMC|IPCA|RNSC| 4 1.10988048731231 response to chemical stimulus, regulation of metabolic process, response to stress
P02511
P04792
Q16082
Q9UJY1
>CCMC_C482|3|CMC|IPCA|RNSC| 4 1.10887377443583 others

P01033
P03956
P08253
P08254
>CCMC_C152|2|CMC|IPCA| 4 1.10807094093611 protein metabolic process
O00548
P46531
P78504
Q04721
>CCMC_C248|2|CMC|HACO| 7 1.10780568556722 regulation of gene expression, regulation of metabolic process
O00257
O95503
O95931
P51784
Q14781
Q93009
Q9HC52
>CCMC_C160|2|CMC|IPCA| 7 1.10460632584627 regulation of metabolic process
O14763
O15519
P48023
P50591
Q13158
Q14790
Q92851
>CHACO_C10667|2|CMC|HACO| 4 1.102285669291 regulation of metabolic process
O14733
Q13387
Q16584
Q9UQF2
>CRNSC_C1|3|CL1|IPCA|RNSC| 10 1.08993043007147 RNA metabolic process
O15234
P38919
P61326
Q86V81

Q92900
Q96Q15
Q9BZI7
Q9H1J1
Q9UBU9
Q9Y5S9
>CHACO_C10081|2|HACO|IPCA| 4 1.08435752482709 DNA metabolic process, response to stress
P18074
P19447
P28715
Q92889
>CRNSC_C300|4|CL1|IPCA|MCL|RNSC| 7 1.0832882333014 others
P15151
P55196
Q15223
Q495A1
Q92692
Q96NY8
Q9NQS3
>CIPCA_C865|2|CMC|IPCA| 4 1.0804812441241 transport
P28288
P33897
P40855
Q9UBJ2
>CCL1_C114|2|CL1|MCL| 5 1.07959893018418 regulation of gene expression, regulation of metabolic process
P26358
Q96T88
Q9UBC3
Q9UJW3
Q9Y6K1
>CIPCA_C50|3|CL1|IPCA|RNSC| 15 1.07748770628069 RNA metabolic process
O15116
O43172
O43395
O95777
P62310

P62312
Q13107
Q15020
Q53GS9
Q7L2J0
Q8WWY3
Q9UK45
Q9Y333
Q9Y4E8
Q9Y4Z0
>CMCL_C143|4|CL1|IPCA|MCL|RNSC|7 1.0718721874484 transport, organelle organization
O14579
P35606
P48444
P53618
P53621
Q9NTJ5
Q9Y678
>CCMC_C259|2|CMC|HACO| 5 1.07104268369296protein metabolic process, transport, RNA metabolic process
P61247
P62241
P62266
P62701
P62913
>CCMC_C802|6|CL1|CMC|HACO|IPCA|MCL|RNSC| 4 1.06785841045049 others

O95670
Q3MIW9
Q6UXA7
Q9UIG4
>CHACO_C14699|3|CMC|HACO|MCL|4 1.06196795957969 transport, small molecule metabolic process
O75947
O75964
P24539
P36542

>CHACO_C13536|2|CMC|HACO| 4 1.06108673043833 others
P02585
P13805
P19237
P48788
>CIPCA_C3474|3|CL1|IPCA|MCL|4 1.06074217740744 small molecule metabolic process
O14521
P21912
P31040
Q99643
>CHACO_C15230|6|CL1|CMC|HACO|IPCA|MCL|RNSC| 4 1.04781885396938 response to chemical stimulus
Q15620
Q15622
Q8WZ84
Q9H209
>CCL1_C476|3|CL1|IPCA|MCL| 4 1.04703413949616 transport
O43525
O43526
P56696
Q9NR82
>CIPCA_C2311|3|CL1|IPCA|MCL|4 1.04587886212739 small molecule metabolic process, regulation of metabolic process, response to stress
O75052
P29474
P29475
P35228
>CMCL_C346|2|CMC|MCL| 4 1.04237901067069 protein metabolic process, response to chemical stimulus, regulation of gene expression, regulation of metabolic process, organelle organization, response to stress
P56524
Q8WUI4
Q9UKV0
Q9UQL6
>CCMC_C673|4|CMC|HACO|IPCA|RNSC| 4 1.0415812096663 others
P04090
Q14641

Q8TDU9
Q9NSD7
>CCMC_C710|5|CL1|CMC|IPCA|MCL|RNSC| 4 1.04034955032291 others
Q86YN6
Q8N8E2
Q96C00
Q9NTW7
>CHACO_C13514|2|CMC|HACO| 5 1.03633251774718protein metabolic process, transport, RNA metabolic process
P46778
P61247
P62424
P62750
P62899
>CRNSC_C109|4|CL1|IPCA|MCL|RNSC| 4 1.03576208108203 response to chemical stimulus
P50552
Q15942
Q8N8S7
Q9UGI8
>CHACO_C15033|5|CL1|CMC|HACO|IPCA|RNSC| 4 1.03556367014369 others

Q8IY31
Q9BW83
Q9NQC8
Q9UG01
>CMCL_C406|2|CMC|MCL| 4 1.03368423139503 others
O95166
Q9GZQ8
Q9H0R8
Q9H492
>CHACO_C11607|2|CMC|HACO| 4 1.0333915244601 RNA metabolic process, response to stress
O95232
P78362
Q07955
Q96SB4
>CRNSC_C313|2|IPCA|RNSC| 9 1.0330741080362 regulation of metabolic

process
O15105
O43541
P36897
P84022
Q13485
Q15796
Q15797
Q9HAU4
Q9HCE7
>CCL1_C304|4|CL1|IPCA|MCL|RNSC| 4 1.0277460992622 regulation of gene expression, regulation of metabolic process
P29374
Q4LE39
Q9H0E3
Q9H7L9
>CRNSC_C336|3|CL1|IPCA|RNSC| 6 1.02442185690842 regulation of metabolic process, response to stress
P29466
Q9C000
Q9HC29
Q9NPP4
Q9ULZ3
Q9Y239
>CCL1_C335|3|CL1|IPCA|MCL| 4 1.02272856673299 protein metabolic process

Q53GT1
Q9P2J3
Q9P2N7
Q9UJP4
>CRNSC_C269|3|CL1|IPCA|RNSC| 4 1.01644863647859 response to stress
Q13438
Q96KS0
Q9GZT9
Q9H6Z9
>CHACO_C14823|2|CMC|HACO| 4 1.01642447718291 response to chemical stimulus
O14879

P09913
P09914
Q13310
>CHACO_C13835|3|CMC|HACO|RNSC| 4 1.01551500712311 others
P09238
P24347
P50281
P51511
>CHACO_C10353|2|CMC|HACO| 4 1.01443349010112 regulation of gene expression, regulation of metabolic process
P13349
P15172
P15173
P23409
>CHACO_C15322|2|CMC|HACO| 7 1.01427126574352 RNA metabolic process
O60563
O60583
P42568
P50750
P51825
Q03111
Q9UHB7
>CHACO_C11386|2|CMC|HACO| 4 1.01307758941638 transport
O15400
Q96AJ9
Q9UEU0
Q9UNK0
>CCMC_C335|2|CMC|HACO| 4 1.0118963502366 regulation of gene expression, regulation of metabolic process
O43439
O75081
Q01196
Q06455
>CRNSC_C28|3|CL1|IPCA|RNSC| 10 1.00626966463108 others
O14817
P05556
P08962
P15529

P21926
P27701
P48509
P60033
Q969P0
Q9P2B2
>CCMC_C298|2|CMC|IPCA| 4 0.998732767038229 organelle organization
P05783
P05787
P08727
Q04695
>CCMC_C353|2|CMC|HACO| 4 0.995516905914543 others
Q96A35
Q9BYD3
Q9NRX2
Q9NX20
>CIPCA_C391|3|CMC|HACO|IPCA| 5 0.990068455569464 transport
O15533
P04439
P30443
P53618
Q9Y678
>CCMC_C539|3|CMC|IPCA|RNSC| 4 0.987852836891692 protein metabolic process
P01040
P04264
P07476
P23490
>CHACO_C10569|2|CMC|HACO| 4 0.982628768076607 transport, RNA metabolic process
Q92797
Q9NZC7
Q9P2I0
Q9UKF6
>CCMC_C297|2|CMC|IPCA| 4 0.978464355567322 transport
P49418
P50570
Q05193

Q99962
>CCMC_C757|5|CMC|HACO|IPCA|MCL|RNSC| 4 0.976136419274133 regulation of metabolic process
O94810
P49758
P49802
P57771
>CCMC_C255|2|CMC|RNSC|4 0.975746028565873 small molecule metabolic process, transport, response to chemical stimulus
P35658
P37198
P49790
P52948
>CCMC_C310|2|CMC|RNSC|4 0.97425290068544 others
O95049
O95832
Q07157
Q9UDY2
>CRNSC_C316|2|IPCA|RNSC| 4 0.973784488717988 small molecule metabolic process
O43837
P50213
P51553
Q96I99
>CHACO_C15269|2|CMC|HACO| 5 0.972435939207604 protein metabolic process, organelle organization, regulation of gene expression, regulation of metabolic process
O15379
P56524
Q8WUI4
Q969S8
Q9UBN7
>CHACO_C11517|2|CMC|HACO| 4 0.950103927867116 RNA metabolic process
P10276
P13631
P19793
P48443

>CHACO_C8054|1|HACO| 6 0.947756001953244 protein metabolic process, cell cycle process, small molecule metabolic process, RNA metabolic process, regulation of metabolic process, response to stress
P28070
P28074
P49720
P49721
Q99436
Q9Y244
>CCMC_C300|2|CMC|MCL| 7 0.944125344953628 regulation of gene expression, regulation of metabolic process
O00221
P19838
P20749
P25963
Q00653
Q04864
Q15653
>CHACO_C8073|1|HACO| 6 0.943583454439403 protein metabolic process, cell cycle process, small molecule metabolic process, RNA metabolic process, regulation of metabolic process, response to stress
P17980
P35998
P43686
P62195
P62333
Q16401
>CCL1_C344|3|CL1|IPCA|RNSC| 5 0.943478309289926 others
P04271
P06703
P08133
P23297
P26447
>CRNSC_C180|4|CL1|IPCA|MCL|RNSC| 4 0.940420053092417 others
P05451
P48304
Q06141
Q86YB8

>CIPCA_C1037|3|CL1|IPCA|MCL|4 0.939676785545457 protein metabolic process, response to stress
O00187
P11226
P48740
Q15485
>CCMC_C699|4|CMC|HACO|IPCA|RNSC| 4 0.938615592871287 response to chemical stimulus
O75093
O94813
Q9HCK4
Q9Y6N7
>CCMC_C808|5|CL1|CMC|HACO|IPCA|RNSC| 4 0.935497554268006 organelle organization
P06899
Q8N257
Q99879
Q99880
>CHACO_C7953|1|HACO| 4 0.935228738750984 regulation of metabolic process, RNA metabolic process
O00268
Q15543
Q15544
Q6P1X5
>CHACO_C13018|2|CMC|HACO| 4 0.93414558566409 response to chemical stimulus
P13501
P32246
P49682
P51681
>CRNSC_C356|4|CMC|HACO|IPCA|RNSC| 4 0.92948712684193 regulation of gene expression, regulation of metabolic process
O75840
O95600
P57682
Q9BXK1
>CHACO_C15293|3|CMC|HACO|IPCA|4 0.921646442993689 response to chemical stimulus

O00626
P51679
P51685
Q92583
>CRNSC_C158|2|IPCA|RNSC| 4 0.921636736347688 small molecule metabolic process
P20338
P20339
P51149
Q15276
>CMCL_C61|4|CL1|IPCA|MCL|RNSC| 10 0.917243722139256 regulation of metabolic process
O00144
O60353
O75084
Q13467
Q14332
Q9H461
Q9NPG1
Q9ULV1
Q9ULW2
Q9UP38
>CMCL_C276|3|CL1|IPCA|MCL| 5 0.915579702447117 RNA metabolic process
O15160
O15446
O95602
Q9GZS1
Q9Y2S0
>CCMC_C582|3|CMC|IPCA|RNSC| 4 0.913425898355373 DNA metabolic process
P31941
P41238
Q9HC16
Q9NRW3
>CCL1_C393|3|CL1|IPCA|RNSC| 4 0.911297145476059 cell cycle process
P43034
Q14203

Q14204
Q9GZM8
>CHACO_C8835|1|HACO| 8 0.90708864619571 protein metabolic process, cell cycle process, small molecule metabolic process, RNA metabolic process, regulation of metabolic process, response to stress
O00232
O00487
O43242
P48556
P51665
Q15008
Q16401
Q9UNM6
>CCMC_C416|2|CMC|HACO| 4 0.905589266650128 regulation of metabolic process
O75143
O75385
Q8IYT8
Q8TDY2
>CRNSC_C72|2|IPCA|RNSC| 6 0.902155407683699 others
P02533
P02538
P04259
P08779
P13647
Q04695
>CMCL_C243|2|IPCA|MCL| 5 0.897126391009056 others
O00501
O14493
O15551
O95471
O95832
>CCMC_C552|3|CMC|IPCA|RNSC| 4 0.883529375778514 regulation of gene expression, regulation of metabolic process
O14640
O14641
O15169
Q92997

>CCMC_C611|3|CMC|MCL|RNSC| 5 0.881391133807938 others
P11055
P12882
P13535
Q9UKX2
Q9Y623
>CHACO_C13572|4|CMC|HACO|IPCA|RNSC| 4 0.880594556852008
response to chemical stimulus
O14904
O14905
Q9GZT5
Q9Y6F9
>CCL1_C405|3|CL1|IPCA|MCL| 4 0.87721478388477 others
O75969
Q15506
Q5JQC9
Q9HAT0
>CMCL_C502|4|CL1|IPCA|MCL|RNSC|4 0.876754819817984 transport
O43747
Q10567
Q9BXS5
Q9H1H9
>CIPCA_C1024|4|CL1|IPCA|MCL|RNSC| 4 0.874407113650682 others
O75970
Q12923
Q9HB19
Q9HB21
>CCL1_C20|2|CL1|RNSC| 6 0.872657740936474 RNA metabolic process
P13984
P29083
P29084
P35269
Q00403
Q9Y5B0
>CHACO_C13510|2|CMC|HACO| 4 0.872506210678493 transport
P02766
P02768
P02787

P10909
>CMCL_C477|4|CL1|IPCA|MCL|RNSC|4 0.872245657843395 others
P51888
P98095
P98160
Q14112
>CCMC_C362|2|CMC|RNSC|6 0.870651605606525 regulation of gene expression, regulation of metabolic process
P03372
P06401
P10275
Q15788
Q92731
Q9Y6Q9
>CRNSC_C301|2|IPCA|RNSC| 7 0.868731331947096 response to chemical stimulus, regulation of gene expression, regulation of metabolic process, response to stress
O00206
O43187
P51617
P58753
Q99836
Q9NWZ3
Q9Y616
>CIPCA_C1017|3|CL1|IPCA|RNSC| 4 0.865804050940834 response to stress
P00813
P29274
P30542
P33765
>CHACO_C11006|2|CMC|HACO| 6 0.864117088241263 others
O95983
Q86YP4
Q8NHZ7
Q8WWY6
Q9BTC8
Q9UBB5
>CHACO_C15360|2|CMC|HACO| 8 0.860564215511469 organelle

organization
O14497
Q86U86
Q8NFD5
Q8TAQ2
Q92785
Q92922
Q969G3
Q96GM5
>CCMC_C372|2|CMC|RNSC|6 0.854886798005064 response to chemical stimulus, regulation of metabolic process
O14543
O15524
O60674
P06213
P35568
Q9Y4H2
>CCMC_C459|2|CMC|HACO| 6 0.853647026959621 regulation of metabolic process
O15392
P98170
Q13489
Q13490
Q96CA5
Q9NR28
>CHACO_C10115|1|HACO| 10 0.84901058465343 regulation of metabolic process
O00231
O00232
O00487
O75832
P51665
P54578
P55036
Q15008
Q9UNM6
Q9Y5K5
>CRNSC_C39|3|IPCA|MCL|RNSC|6 0.848814488674389 response to chemical

stimulus
P09341
P19875
P19876
P42830
P78556
P80162
>CHACO_C15020|3|CMC|HACO|IPCA|4 0.845842814410761 organelle organization
P0C0S8
P33778
P68431
Q16777
>CCMC_C467|2|CMC|RNSC|4 0.841964862584231 regulation of metabolic process
P08476
P09529
P27037
P36896
>CCL1_C340|2|CL1|IPCA| 4 0.838477855417458 regulation of gene expression, regulation of metabolic process, RNA metabolic process
P11474
P51843
P62508
Q13285
>CHACO_C8810|1|HACO| 5 0.8384131466575 cell cycle process
O43683
O95229
Q9BZD4
Q9H081
Q9H410
>CHACO_C8258|1|HACO| 4 0.835128046248538 protein metabolic process, transport, RNA metabolic process
P46777
P62081
P62241
P62913
>CHACO_C11910|2|CMC|HACO| 4 0.834268513688153 regulation of

metabolic process, response to stress
O60603
Q9NR96
Q9NR97
Q9Y2C9
>CRNSC_C233|2|MCL|RNSC| 16 0.833655519264696 others
O00506
P30154
Q14BN4
Q5VSL9
Q8WZ74
Q9BRV8
Q9BUL8
Q9NRL3
Q9NVK5
Q9P289
Q9P2B4
Q9UGI0
Q9ULQ0
Q9Y228
Q9Y3A3
Q9Y6E0
>CRNSC_C291|2|IPCA|RNSC| 4 0.831605628046886 small molecule metabolic process, regulation of metabolic process
O00330
P09622
P10515
Q15119
>CHACO_C9456|1|HACO| 5 0.831384819061789 RNA metabolic process
Q01780
Q5RKV6
Q8TF46
Q96B26
Q9NQT4
>CHACO_C14492|4|CMC|HACO|IPCA|RNSC| 4 0.830892356346011 others

Q86SS6
Q8NBV8

Q9BQS2
Q9BT88
>CRNSC_C199|3|CMC|IPCA|RNSC| 4 0.827998570490767 RNA metabolic process
O43660
P52294
Q8WYA6
Q9UMS4
>CCMC_C436|2|CMC|IPCA| 7 0.824903581149669 response to chemical stimulus, organelle organization
O43521
P10415
Q07812
Q07820
Q13794
Q16611
Q92843
>CHACO_C13855|4|CMC|HACO|IPCA|RNSC| 4 0.823055879068651 others

Q5EBM0
Q5K651
Q8IVG5
Q9P2E3
>CIPCA_C1850|3|CL1|IPCA|MCL|4 0.820044642590524 others
Q96HJ5
Q96JQ5
Q9GZW8
Q9H2W1
>CHACO_C13507|2|CMC|HACO| 4 0.81837768018344 regulation of gene expression, regulation of metabolic process
P28356
P31277
P35452
Q03828
>CCMC_C490|2|CMC|MCL| 4 0.817992446873004 protein metabolic process

O43294
P49023

Q05209
Q14289
>CRNSC_C4|3|CL1|IPCA|RNSC| 5 0.812647549432872 response to chemical stimulus
P01583
P01584
P14778
P18510
P27930
>CHACO_C10111|1|HACO| 7 0.812406632927977 RNA metabolic process
Q93074
Q96RN5
Q9BUE0
Q9H944
Q9NVC6
Q9NWA0
Q9UHV7
>CMCL_C446|3|IPCA|MCL|RNSC| 4 0.811103825334222 regulation of metabolic process, response to stress
Q5S007
Q99497
Q9BXM7
Q9NQ11
>CHACO_C10530|2|CMC|HACO| 4 0.80943362826061 organelle organization
P35606
P53618
P53621
Q9BVK6
>CHACO_C11723|2|CMC|HACO| 4 0.808315216500052 regulation of gene expression, regulation of metabolic process
O60353
O75084
Q13467
Q9H461
>CHACO_C10850|2|HACO|RNSC| 4 0.804350401401982 others
Q13362
Q14738
Q15172

Q5FBB7
>CCMC_C13|1|CMC| 6 0.804 small molecule metabolic process
O43678
O75306
O75489
P19404
P28331
P49821
>CIPCA_C1082|2|CL1|IPCA| 4 0.796595028335492 response to chemical stimulus, response to stress
P05107
P05362
P11215
P20701
>CCMC_C430|2|CMC|IPCA| 4 0.79503478065956 response to chemical stimulus, regulation of metabolic process, organelle organization
P60953
P63000
Q13153
Q14155
>CCMC_C18|1|CMC| 6 0.793 protein metabolic process, regulation of metabolic process
O00232
P55036
P60900
Q15008
Q9UNM6
Q9Y5K5
>CHACO_C14904|3|CMC|HACO|RNSC| 4 0.789133234447356 small molecule metabolic process
P11488
P16499
P19087
P35913
>CCMC_C653|3|CMC|IPCA|RNSC| 4 0.788637166663951 others
P02545
P20700
P42166

P42167
>CRNSC_C22|4|CL1|IPCA|MCL|RNSC|4 0.788020130165578 others
O75487
P35052
P78333
Q9Y625
>CHACO_C10522|1|HACO| 6 0.784088494194509 response to stress
O14818
P25788
P25789
P28066
P60900
Q9BQ83
